# A SNP resource for Douglas-fir: *de novo* transcriptome assembly and SNP detection and validation

**DOI:** 10.1186/1471-2164-14-137

**Published:** 2013-02-28

**Authors:** Glenn T Howe, Jianbin Yu, Brian Knaus, Richard Cronn, Scott Kolpak, Peter Dolan, W Walter Lorenz, Jeffrey FD Dean

**Affiliations:** 1 Department of Forest Ecosystems and Society, Oregon State University, Corvallis, Oregon, 97331, USA; 2 Current address, DuPont Pioneer International, Willmar, Minnesota, 56201, USA; 3 Pacific Northwest Research Station, USDA Forest Service, Corvallis, Oregon, 97331, USA; 4 Department of Mathematics, University of Minnesota, Morris, MN, USA; 5 Warnell School of Forestry and Natural Resources, University of Georgia, Athens, Georgia, 30602, USA

## Abstract

**Background:**

Douglas-fir (*Pseudotsuga menziesii*), one of the most economically and ecologically important tree species in the world, also has one of the largest tree breeding programs. Although the coastal and interior varieties of Douglas-fir (vars. *menziesii* and *glauca*) are native to North America, the coastal variety is also widely planted for timber production in Europe, New Zealand, Australia, and Chile. Our main goal was to develop a SNP resource large enough to facilitate genomic selection in Douglas-fir breeding programs. To accomplish this, we developed a 454-based reference transcriptome for coastal Douglas-fir, annotated and evaluated the quality of the reference, identified putative SNPs, and then validated a sample of those SNPs using the Illumina Infinium genotyping platform.

**Results:**

We assembled a reference transcriptome consisting of 25,002 isogroups (unique gene models) and 102,623 singletons from 2.76 million 454 and Sanger cDNA sequences from coastal Douglas-fir. We identified 278,979 unique SNPs by mapping the 454 and Sanger sequences to the reference, and by mapping four datasets of Illumina cDNA sequences from multiple seed sources, genotypes, and tissues. The Illumina datasets represented coastal Douglas-fir (64.00 and 13.41 million reads), interior Douglas-fir (80.45 million reads), and a Yakima population similar to interior Douglas-fir (8.99 million reads). We assayed 8067 SNPs on 260 trees using an Illumina Infinium SNP genotyping array. Of these SNPs, 5847 (72.5%) were called successfully and were polymorphic.

**Conclusions:**

Based on our validation efficiency, our SNP database may contain as many as ~200,000 true SNPs, and as many as ~69,000 SNPs that could be genotyped at ~20,000 gene loci using an Infinium II array—more SNPs than are needed to use genomic selection in tree breeding programs. Ultimately, these genomic resources will enhance Douglas-fir breeding and allow us to better understand landscape-scale patterns of genetic variation and potential responses to climate change.

## Background

The availability of high-throughput sequencing methods has led to the discovery of thousands to millions of single nucleotide polymorphisms (SNPs) in diverse organisms, particularly humans, model experimental organisms, and agriculturally important plants and animals. Combined with high-throughput genotyping platforms, SNP markers are having substantial impacts on human medicine as well as plant and animal breeding [1-3]. They are also being used to provide detailed insights into the population genetics of natural populations, and are likely to help elucidate the functional basis of simply inherited traits. In addition, they are frequently cited as the solution for understanding the explicit genetic basis of quantitative traits [4], although prospects for the latter remain uncertain [5].

Our main goal was to develop and test a large number of SNP markers for Douglas-fir (*Pseudotsuga menziesii* (Mirb.) Franco) that could be used to enhance and accelerate Douglas-fir breeding via genomic selection. Tree breeders typically make breeding decisions based on an individual’s breeding value, which is the average performance of an individual’s offspring. Currently, breeding values are estimated from measurements made in progeny tests containing thousands to tens of thousands of trees. Genomic selection, or whole-genome marker-assisted selection [6], could revolutionize tree breeding by allowing breeders to dramatically reduce the generation interval and extent of progeny testing. Genomic selection has been widely adopted in livestock breeding [7], where empirical studies suggest that accuracies of genomic selection are often 70% or more, compared to accuracies of 30 to 40% for breeding values estimated from parental performance, and accuracies of about 85% for breeding values estimated from progeny testing, which is both time-consuming and costly [3]. However, these encouraging results required SNP resources consisting of thousands to tens of thousands of SNPs—numbers that far exceed what is available for Douglas-fir. In addition to genomic selection, SNP markers are expected to replace simple sequence repeats (SSRs) for routine, automated uses of markers for other breeding program applications. The high variability of SSR markers makes them ideal for many applications, but automated marker scoring is often challenging. In seed orchards, genetic markers (mostly SSRs) are routinely used to confirm the identity of seed orchard trees, measure pollen contamination, assess the effectiveness of pollen management techniques, measure and manage inbreeding and genetic diversity, determine parental contributions to open-pollinated seedlots (i.e., progeny populations), and verify seedlot integrity [8,9]. Highly informative genetic markers may also allow breeders to combine simple, cost-effective mating designs (e.g., polymix or open-pollinated designs) with parental analysis to reduce breeding costs, speed breeding progress, and increase genetic gains [10,11].

Douglas-fir is one of the most ecologically and economically important tree species in the world. It occupies diverse habitats from central British Columbia to Mexico, and from the Pacific Ocean to the eastern slopes of the Rocky Mountains. In the Pacific Northwest, coastal Douglas-fir (var. *menziesii*) forms ancient forests that serve as key habitats for endangered species, and are widely grown in plantations that form the foundation of a multi-billion dollar forest products industry. In the Rocky Mountains, the interior or Rocky Mountain variety (var. *glauca* (Beissn.) Franco) occupies mostly drier and colder sites, and has a more varied impact on the ecology and economy of the region. In Mexico, Douglas-fir exists as widely dispersed ‘sky-island’ populations that are typically considered extensions of var. *glauca*, but may deserve their own varietal status [12,13]. Overall, Douglas-fir is ecologically, physiologically, and genetically diverse, within and among varieties (reviewed in [14]). Because of its economic importance, Douglas-fir has one of the largest tree breeding programs in the world. The Northwest Tree Improvement Cooperative program for coastal Douglas-fir has nearly 4 million tested trees, including more than 31,000 first-generation parents tested on 1,016 progeny test sites, and 2,980 second-cycle crosses tested on 129 sites [14] (K. Jayawickrama, personal communication). Smaller breeding programs exist for interior Douglas-fir in the United States and Canada (reviewed in [14]). In coastal Douglas-fir, breeding focuses on improving growth, stem form, and wood properties while maintaining climatic adaptability.

Our primary goal was to greatly expand the SNP resources for Douglas-fir beyond the 200–300 validated SNPs that were currently available [15]. Therefore, we combined two high-throughput sequencing technologies (454 pyrosequencing and Illumina sequencing-by-synthesis) to sequence the transcriptomes of diverse tissues and Douglas-fir genotypes. Our objectives were to (1) develop a reference transcriptome for coastal Douglas-fir by combining existing Sanger sequences with new 454 sequences, (2) annotate and evaluate the quality of the reference transcriptome, (3) map 454 and Illumina short-read sequences to the reference and identify SNPs, and (4) construct and test a high-density Infinium genotyping array. In addition to the SNP markers we developed, our reference transcriptome will facilitate studies of gene expression and function, and will aid efforts to assemble and annotate reference genome sequences of Douglas-fir and other conifers (http://pinegenome.org/pinerefseq/).

## Results

### Pre-assembly sequence processing for the reference transcriptome

**Figure 1:**
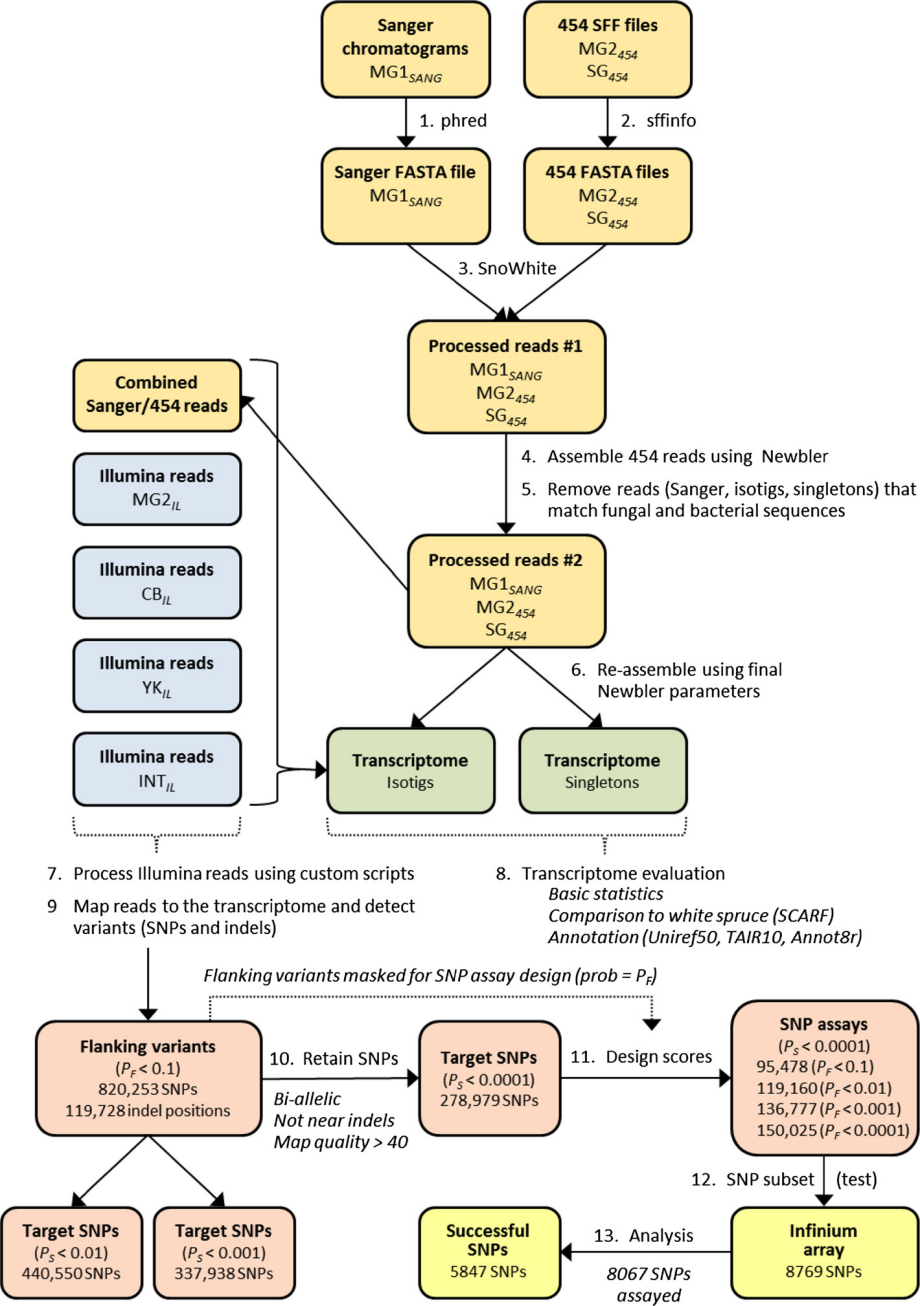
**Strategy for assembling the Douglas-fir reference transcriptome and detecting SNPs.** We used one Sanger sequence dataset (MG1_*SANG*_) and two 454 sequence datasets (MG2_*454*_ and SG_*454*_) to assemble the reference transcriptome. We then used these same datasets plus four Illumina short read datasets (MG2_*IL*_, CB_*IL*_, YK_*IL*_, INT_*IL*_) to detect flanking variants. Orange boxes represent Sanger and 454 datasets, blue boxes represent Illumina short-read datasets, green boxes represent the reference transcriptome, red boxes represent SNP filtering steps, and yellow boxes represent SNP genotyping and analytical steps. The number of SNPs for which Infinium genotyping assays were successfully designed (Assay Design Tool score ≥ 0.6) depends on the probability used for filtering the target SNPs (*P*_*S*_*<* 0.01, 0.001, and 0.0001) and the probability used to mask nucleotides in the flanking regions (*P*_*F*_ = 0.1, 0.01, 0.001, and 0.0001). Larger *P*_*F*_ values resulted in more flanking variants and fewer target SNPs with successful designs.

We used long reads from three datasets as the basis for *de novo* assembly of a reference transcriptome for coastal Douglas-fir. Prior to the final assembly, we cleaned and filtered these datasets as shown in Figure 1 (Steps 1–5). These datasets included 454 sequences from a single genotype (SG_*454*_ = 1.241 M reads) and sequences from two multi-genotype pools produced using 454 pyrosequencing (MG2_*454*_ = 1.709 M reads) and Sanger sequencing (MG1_*SANG*_ = 12,157 reads). Our initial pool of 2.96 × 10^6^ reads was reduced to 2.78 × 10^6^ reads after filtering using the SnoWhite pipeline (Table 1). The percentage of filtered sequences was substantially smaller for the normalized than for the non-normalized 454 dataset (2.4% for MG2_*454*_ versus 11.2% for SG_*454*_), and this effect was most pronounced for the rRNA and retrotransposon-like sequences (Table 1). After removing additional fungal and bacterial sequences, and excluding reads shorter than 50 nt, 2.76 × 10^6^ sequences were available to assemble the reference transcriptome (Table 2).

**Table 1:** Sequence datasets used to construct the Douglas-fir reference transcriptome*

**Plant materials (dataset ID) *Collection information***	**Method**^**† **^***cDNA library***	**Total reads in dataset (%)**	**Number of reads filtered from the input dataset (% of library total)**					
			**Short or low-quality**	**Adapter or vector**	**Chloro-plast**	**Mitochon-drial**	**rRNA**	**Retro-transposon**
Multi-genotype #1 (MG1_*SANG*_)	Sanger	12,157 (100)	57 (0.47)	0 (0.00)	2 (0.02)	2 (0.02)	0 (0.00)	1 (0.01)
* Cold season*	*Normalized*							
* Greenhouse*	*Non-normalized*							
Multi-genotype #2 (MG2_*454*_)	GS-FLX Titanium	1,709,211 (100)	6649 (0.39)	1893 (0.11)	8570 (0.50)	5519 (0.32)	7264 (0.42)	11,114 (0.65)
* Cold and warm seasons*	*Normalized*							
Single-genotype (SG_*454*_)	GS-FLX Titanium	1,241,260 (100)	6582 (0.53)	1826 (0.15)	11,070 (0.89)	10,463 (0.84)	86,828 (7.00)	21,849 (1.76)
* July 8, 2008*	*Non-normalized*							
All datasets		2,962,628 (100)	13,288 (0.45)	3719 (0.13)	19,642 (0.66)	15,984 (0.54)	94,092 (3.18)	32,964 (1.11)

* For each dataset, the numbers of reads filtered using the SnoWhite pipeline (Figure 1, Step 3) are shown by sequence type.

^†^ GS-FLX Titanium is the Roche 454 sequencing platform.

**Table 2:** Characteristics of the Douglas-fir transcriptome assembly using Newbler v2.3

		**Length (nt)**			
**Statistic**	**Number**	**Mean**	**Median**	**N50**	**Total**
Reads used by Newbler^*^	2,764,549	360	392	416	996,614,802
Reads assembled by Newbler^†^	2,544,087	364	394	416	925,577,338
Isotigs^§^	38,589	1390	1141	1883	53,622,767
Isogroups	25,002	1443	1181	1864	36,069,331
Isogroups with 1 isotig (I1)	18,774	1334	1053	1750	25,046,862
Isogroups with >1 isotig (IM)^‡^	6228	1770	1547	2141	11,022,469
Singletons	102,623	356	384	413	36,504,221
Total (isogroups + singletons)	127,625	569	413	517	72,573,552

^*^ The input number of reads is less than the total in Table 1 (2.96 × 10^6^) because reads shorter than 50 nt were excluded. Statistics were calculated using the sequences actually used in the assembly after applying a default minimum length of 40 for reads trimmed by Newbler.

^†^ Includes reads that assembled as complete reads or as partial reads.

^§^ Isotigs ≥ 200 nt were deposited at DDBJ/EMBL/GenBank under accession GAEK01000000.

^‡^ Statistics for the IM isogroups were calculated using the longest isotig in each isogroup.

### Assembly of the reference transcriptome

**Figure 2:**
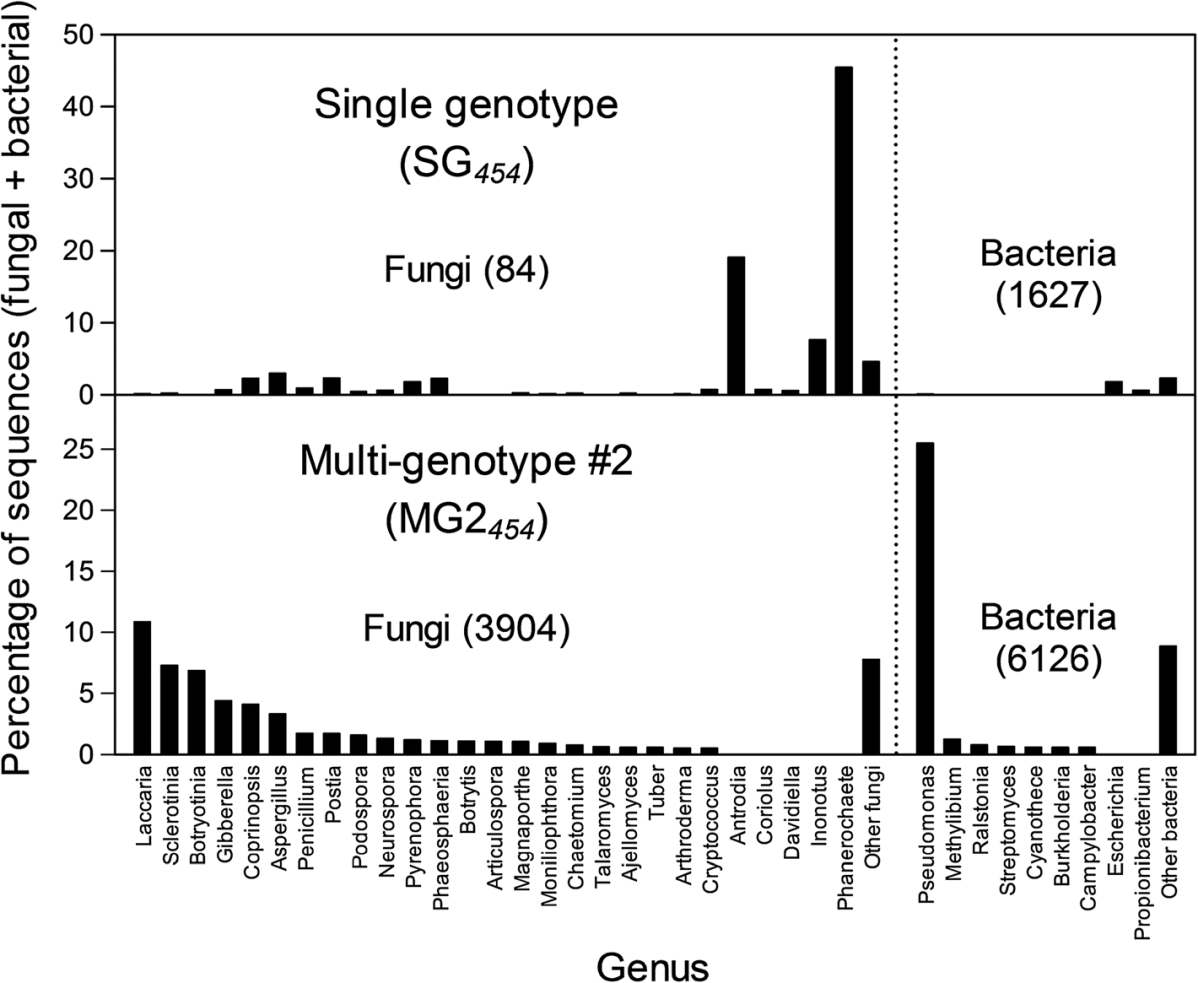
**Taxonomic distributions of Douglas-fir sequences identified as bacterial or fungal contaminants.** We used preliminary assemblies of the SG_*454*_ and MG2_*454*_ datasets and BLAST searches to identify isotigs and singletons resulting from bacterial or fungal contamination (see Methods). Reads corresponding to these singletons and isotigs were removed prior to the final assembly. Numbers in parentheses are the total number of sequences (isotigs and singletons) in each category.

In this section, we describe the preliminary and final assemblies of the reference transcriptome (Figure 1, Steps 4–6), and analyses we used to infer the orientation of the resulting isotigs and singletons. Different assembly parameters resulted in few differences in the number of resulting isogroups (overlap length = 35 or 45; overlap identity = 82 to 98%; overlap difference score = −2 or −6). In particular, there was almost no increase in the total number of isogroups when the overlap identity was increased from 82% to 90%, and only a slight increase from 90% to 98%. The final *de novo* assembly was constructed using a minimum overlap length of 45 nt, minimum overlap identity of 96%, and an alignment difference score of −6. However, before conducting the final assembly, we assembled the 454 datasets (MG2_*454*_ and SG_*454*_) separately, and then used BLASTN to compare the resulting isotigs and singletons to a series of databases to identify and remove sequences from contaminating fungal and bacterial organisms (Figure 2). After the final assembly, we used Vmatch to eliminate redundant sequences from 40,010 assembled isotigs, resulting in 38,589 non-redundant isotigs with an average length of 1,390 nt and N50 of 1,883 nt (Table 2). The resulting reference transcriptome consisted of 25,002 isogroups (unique gene models) and 102,623 singletons. Of these 25,002 isogroups, 18,744 were represented by a single isotig (transcript variant), and are inferred to correspond to a single transcript. These isogroups and isotigs are referred to as the ‘I1’ (Isogroups with 1 isotig) subset in the following analyses. The remaining 6,228 isogroups were represented by multiple isotigs, which suggests they represent alternatively spliced transcripts from the same gene. These isogroups and isotigs are subsequently referred to as the ‘IM’ (Isogroups with Multiple isotigs) subset. The reference transcriptome (i.e., 37,177 isotigs ≥ 200 nt) has been deposited at DDBJ/EMBL/GenBank under accession GAEK01000000. The characteristics of the transcriptome isotigs and singletons are described in Additional files 1 and 2.

Mapping of strand-oriented reads from the CB_*IL*_ and YK_*IL*_ libraries allowed us to infer the orientations of 73.4% of the isotigs and 9.5% of the singletons (Additional file 1: Tables S1 and S3). The orientations of the remaining isotigs and singletons were ambiguous because the binomial test was non-significant or no data were available (i.e., no Illumina reads were successfully mapped). Other assembly characteristics are reported in Table 2.

### Comparison to white spruce and loblolly pine

To evaluate our assembled isotigs, we compared them to a well characterized set of white spruce unigenes (Figure 1, Step 8). Isotigs with clear interpretable relationships to these unigenes were assigned high confidence scores, and were preferentially included on the SNP array. We categorized the isotigs into confidence classes (C1-C7) based on their relationships to the white spruce unigenes described by Rigault et al. [16] (Table 3). Lower numbers represent simpler relationships and (hypothetically) greater confidence that the assembly is correct. Although the percentages of unmatched isotigs (Class C7) were roughly equal for the two subsets (I1 = 28% and IM = 24%), the other classes differed dramatically between the I1 and IM subsets (Table 3). This reflects the more complex relationships that are possible for isogroups with multiple isotigs (IM subset), and shows how this leads to generally lower confidence scores for this group.

**Table 3:** **Comparison between Douglas-fir isotigs and white spruce unigenes**[16]

					**Number of isotigs**		
Class^*^	**No. of WS matches**^†^	**Do other DF match the same WS?**^**§**^	**Do other matching DF overlap?**^**‡**^	**Isotig confidence**^#^	**I1 subset (1 isotig per isogroup) (18,774)**	**IM subset (>1 isotig per isogroup) (19,815)**	**Example visual representations**^**@**^
C1	1	No	-	Highest	5140	261	
C2	2+	No	-	Higher	896	88	
C3	1	Yes	No	Higher	1767	577	
C4	2+	Yes	No	Medium	586	159	
C5	1	Yes	Yes	Lower	1736	6974	
C6	2+	Yes	Yes	Lowest	3405	7040	
	**Subtotal**	-	-	-	**13,530**	**15,099**	
C7	No matches	-	-	Unknown	5244	4716	

^*^Douglas-fir (DF) isotigs were categorized into seven classes (C1-C7) and three levels of confidence based on their relationships to white spruce (WS) contigs using the SCARF program [68].

^†^Number of white spruce contigs that matched the Douglas-fir query.

^§^‘Yes’ indicates that at least one non-query isotig also matched the same white spruce contig.

^‡^‘Yes’ indicates that the query and at least one non-query isotig matched the same region of the white spruce contig (overlapped).

^#^Subjective level of confidence in the isotig assembly based on the information presented in columns 2–4.

^@^Cross-hatched bars represent white spruce contigs, black bars represent query Douglas-fir isotigs, and white bars represent non-query Douglas-fir isotigs.

When we conducted the same SCARF analysis using 35,550 loblolly pine contigs, the results were nearly identical to those of white spruce (data not shown). For example, the correlation between the numbers of isotigs in each confidence class was 0.96 between spruce and pine (i.e., 0.96 for both the I1 and IM isotigs). In addition, 67% of the no-hit isotigs found using spruce as the reference (n = 9960) were also no-hits using pine as the reference (n = 6651). Conversely, 80% of the no-hit isotigs found using pine as the reference (n = 8293) were also no-hits using spruce (n = 6651).

### Annotation

We annotated the isotigs and singletons (Figure 1, Step 8), and then used this information to select SNPs for the SNP array. For all three protein databases, the percentages of sequences with matches were highest for the I1 subset, moderate for the IM subset, and lowest for the singletons (S subset) (Table 4). The Annot8r annotation tool creates subsets of selected UniProt databases that only include protein sequences with GO, EC, or KEGG annotations. Therefore, in contrast to the results from Annot8r, many of the proteins in the Uniref50 database, and some of the proteins in the TAIR10 database have unknown functions. Thus, the results from Annot8r provide the percentages of Douglas-fir sequences that can be annotated by function (62.5% for the I1 subset, 46.0% for the IM subset, and 14.5% for the singletons). We subsequently used these annotations to target SNPs associated with growth, phenological traits, stress resistance, or adaptation to temperature or drought. In contrast, the distribution of matches among taxonomic groups did not differ substantially among subsets I1, IM, and S (Table 5). Small percentages (I1 = 0.89%, IM = 0.35%, S = 3.55%) of assembled Douglas-fir sequences matched fungal, bacterial, and viral sequences at an E-value < 10^-5^, which is greater than the much more stringent 10^-10^ E-value we used to identify contaminating isotigs and singletons during the filtering that preceded our final assembly.

**Table 4:** Numbers and percentages of Douglas-fir sequences with matches to sequences in three protein databases*

	**Isogroups (25,002)**^**†**^				**Singletons (102,623)**^**§**^	
	**Isogroups with 1 isotig (I1 = 18,774)**		**Isogroups with >1 isotig (IM = 6228)**		**Singletons (S = 102,623)**	
**Database**	**Number**	**Percent**	**Number**	**Percent**	**Number**	**Percent**
Uniref50	15,054	80.2	3446	55.3	25,757	25.1
TAIR10	13,749	73.2	3260	52.3	15,907	15.5
Annot8r	11,733	62.5	2862	46.0	14,836	14.5

^*^Matches were recorded for isogroups and singletons at a tBLASTX E-value < 10^-5^.

^†^Isogroups are Newbler v2.3 isogroups. For the isogroups with more than 1 isotig (IM subset), a hit was counted only if all isotigs matched the same protein in the database.

^§^Singletons are 454 reads that did not assemble with any other reads.

**Table 5:** Numbers and percentages of Douglas-fir sequences with matches to sequences in the Uniref50 protein database*

	**Isogroups (25,002)**^**†**^				**Singletons (102,623)**^**§**^	
	**Isogroups with 1 isotig (I1 = 18,774)**		**Isogroups with >1 isotig (IM = 6228)**		**Singletons (S = 102,623)**	
**Taxonomic category**	**Number**	**Percent of matches**	**Number**	**Percent of matches**	**Number**	**Percent of matches**
Conifers	4088	27.16	1073	31.14	6486	25.18
Other plants	9713	64.52	2047	59.40	16,061	62.36
Other Eukaryotes	582	3.87	182	5.28	658	2.55
Invertebrates	487	3.24	120	3.48	1087	4.22
Bacteria	123	0.82	8	0.23	830	3.22
Environmental	21	0.14	6	0.17	37	0.14
Vertebrates	17	0.11	6	0.17	92	0.36
Fungi	19	0.13	4	0.12	487	1.89
Viruses	4	0.03	0	0.00	19	0.07
**Total matches**	**15,054**	**100.00**	**3446**	**100.00**	**25,757**	**100.00**
**Unmatched**	**3720**	**-**	**2782**	**-**	**76,866**	**-**
**Percent matched**	**80.2**	**-**	**55.3**	**-**	**25.1**	**-**

^*^Matches are grouped by taxonomic affiliation and percentages are relative to the total number of matches (tBLASTX E-value < 10^-5^). Numbers of input Douglas-fir sequences are in parentheses.

^†^Isogroups are Newbler v2.3 isogroups. For the isogroups with more than 1 isotig (IM subset), a hit was counted only if all isotigs matched the same protein in the database.

^§^Singletons are 454 reads that did not assemble with any other reads.

**Figure 3:**
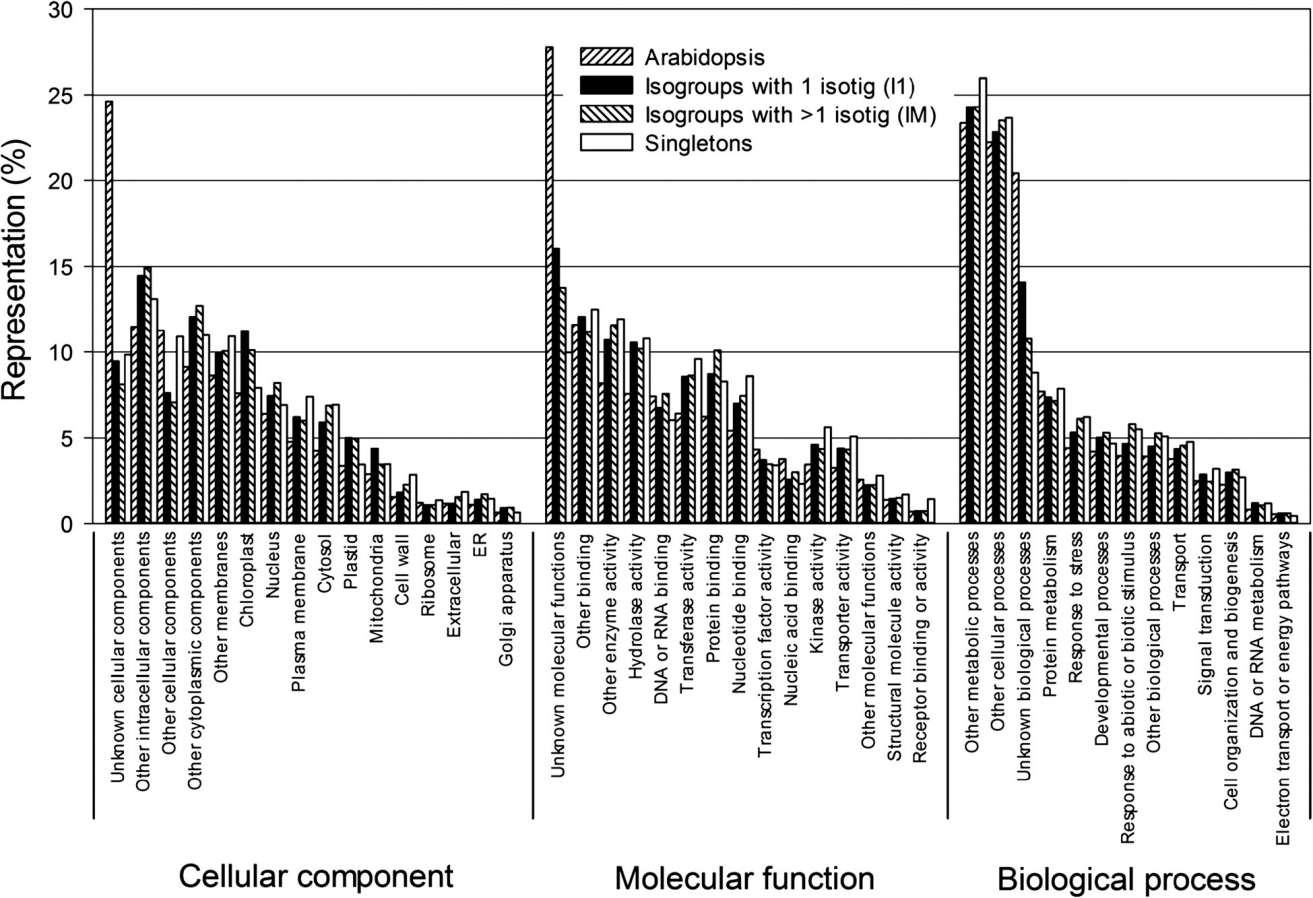
**Distributions of Douglas-fir sequences and *Arabidopsis *genes by GO slim terms.** Distributions are shown for *Arabidopsis* genes (TAIR10 accessions), two types of Douglas-fir isogroups (I1 subset = isogroups with one isotig and IM subset = isogroups with more than one isotig), and Douglas-fir singletons.

The differences in the distributions of GO slim classifications among the three types of Douglas-fir sequences (I1, IM, and S) were small (Figure 3). Compared to Douglas-fir, many more *Arabidopsis* sequences fell into the “unknown cellular components” and “unknown molecular functions” classes. This indicates that Douglas-fir sequences were less likely to match these classes of *Arabidopsis* sequences than others, suggesting that they tend to exhibit species-specific characteristics (i.e., are more highly diverged or absent from Douglas-fir). Presumably, many of the unmatched Douglas-fir genes would fall into these GO slim classes had we used a less stringent E-value.

### SNP detection

Two criteria are important for selecting SNPs for an Infinium genotyping array. First, the target SNP should have a high probability (i.e., low P-value, *P*_S_) of being a true variant. Second, the target SNP should have no variants in its flanking sequences where the genotyping primers must hybridize. Therefore, the P-values for flanking variants (SNPs and indels, *P*_F_) should also be considered. A high SNP conversion rate is expected when a very high P-value (permissive probability threshold) is used for flanking variants, and a very low P-value (stringent probability threshold) is used for target SNPs. However, this approach will dramatically reduce the number of SNPs that can be detected and assayed. In this section, we describe how we filtered all potential target SNPs based on *P*_S_, *P*_F_, and other criteria (Figure 1, Steps 9–10).

We used a permissive probability threshold (*P*_F_ = 0.10) to detect potential SNPs and indels in the flanking regions of target SNPs (Figure 1, Step 9). These positions were then excluded (masked) from consideration when the genotyping primers were designed. Out of a total assembly of 72.6 × 10^6^ nucleotides, we masked 820,253 SNPs and 119,728 indel positions. In the subsequent filtering step to identify target SNPs, we identified bi-allelic SNPs that were not near high-quality indels (i.e., indels with scores ≥ 25), had a mapping quality score > 40 in at least one dataset, and target SNP probabilities (*P*_S_) of < 10^-2^, 10^-3^, or 10^-4^ in at least one dataset (Figure 1, Step 10). For the most stringent (10^-4^) level of probability, these criteria resulted in 278,979 potential SNPs (Additional file 3), 183,380 of which were detected in more than one dataset (Table 6). Many SNPs were detected in both the coastal and interior datasets—151,014 shared SNPs in 17,361 isogroups. On average, these shared SNPs represented 74% of all coastal SNPs and 67% of all interior SNPs. Not surprisingly, more SNPs were detected in the larger datasets (Table 6).

**Table 6:** **Numbers of potential SNPs detected in Douglas-fir using an individual dataset probability value of 10**^**-4**^

			**No. of reads (× 10**^**6**^**)**	No. of unique or shared SNPs^*^				
**Plant materials (dataset ID)**	**Seed source**	**Sequencing platform**		**Unique**	**Coastal**	**Yakima**	**Interior**	**Total**^**†**^
				**--------------- All isotigs (1 isotig/isogroup (I1)) --------------**				
Multi-genotype #1 (MG1_*SANG*_)	Coastal	Sanger	2.77	3982 (2606)	101,089 (85,635)	29,922 (25,523)	81,633 (69,109)	107,884 (90,487)
Multi-genotype #2 (MG2_*454)*_	Coastal	Roche 454						
Single-genotype (SG_*454*_)	Coastal	Roche 454						
Multi-genotype #2 (MG2_*IL*_)	Coastal	Illumina	64.00	18,694 (15,617)	192,693 (162,560)	41,952 (35,700)	146,242 (123,503)	192,693 (162,560)
Coos Bay (CB_*IL*_)	Coastal	Illumina	13.41	1044 (895)	66,304 (56,547)	29,051 (24,703)	53,275 (45,437)	66,304 (56,547)
Yakima (YK_*IL*_)	Yakima	Illumina	8.99	638 (545)	43,066 (36,621)	-	40,840 (34,750)	47,573 (40,505)
Interior (INT_*IL*_)	Interior	Illumina	80.45	71,241 (61,334)	151,014 (127,403)	40,840 (34,750)	-	226,124 (192,076)
**Total**			**169.62**					

^*^The number of unique SNPs and the number of SNPs shared in other datasets of the coastal, Yakima, and interior seed sources are presented for all isogroups (I1 + IM) and for the 1 isotig per isogroup subset (I1) (in parentheses). The total number of unique SNPs detected in all datasets was 278,979.

^†^SNP totals are not the sums of the values in the same row because SNPs are double-counted. For example, we detected 66,304 SNPs in the CB_*IL*_ dataset, 29,051 of which were detected in the YK_*IL*_ dataset and 53,275 of which were detected in the INT_*IL*_ dataset.

### SNP array

In this section, we describe other criteria, including Infinium design scores, which were used to select the final set of SNPs to test on the genotyping array. Design scores are values generated by the Infinium Assay Design Tool that are associated with the performance of SNP assays. Design scores could be generated for only 34% (95,478/278,979) of the target SNPs submitted to Illumina (Figure 1, Step 11), primarily because of the permissive probability threshold (*P*_F_ = 0.10) used for calling variants in the flanking sequences. That is, assays were not possible for 66% of the target SNPs because of flanking SNPs and indels in the assay design region (failure code 106). We then selected 8769 SNPs to test using an Infinium genotyping array (Figure 1, Step 12) [18]. Selection criteria included differential expression, annotations, target SNP probabilities, minor allele frequencies (MAF), Illumina design scores, and SNP array capacity. Of the 8769 attempted SNPs, only 8067 (92%) were assayed because of the normal loss of bead types that occurs during array manufacture (Figure 1, Step 13). Of these, 7256 SNPs had call frequencies ≥ 85%, and 5847 of these were polymorphic in a sample of 260 trees (i.e., successful SNPs in Figure 1 and Table 7). Of the 5847 successful SNPs, 263 (4.5%) had significant deviations from Hardy-Weinberg equilibrium (HWE). The characteristics of the successfully called and polymorphic SNPs are described in Additional file 4 and summarized in Table 8.

**Table 7:** Douglas-fir SNPs detected using an Illumina Infinium SNP array (n = 260 trees)

		No. of SNPs in category/No. of SNPs attempted or assayed (%)^*^	
SNP category	Number of SNPs	Attempted (n=8769)	Assayed (n=8067)
SNPs attempted	8769	100.0	108.7
SNPs assayed	8067	92.0	100.0
Called SNPs (call frequency > 0.85)^†^	7256	82.7	89.9
Called SNPs that are polymorphic (MAF > 0)	5847	66.7	72.5
Percent of called SNPs that are polymorphic (5847/7256) = 80.6			

^*^The number of SNPs in each category is expressed as a percentage of the total number of SNPs attempted (n = 8769) and number of SNPs successfully assayed on the array (n = 8067).

^†^Successful calls are those with a GenCall score ≥ 0.15 [19].

**Table 8:** Characteristics of 5847 successful SNPs based on data from an Illumina Infinium SNP array^*^

**SNP characteristic**	**Mean**	**Median**	**Range**
GenTrain score	0.81	0.84	0.35-0.98
GC50 score (median GenCall score)	0.78	0.87	0.15-0.99
Call frequency^†^	0.99	1.00	0.85-1.00
Minor allele frequency (MAF)	0.24	0.24	0.002-0.5
Heterozygosity (observed)	0.33	0.36	0.00-1.00
Heterozygosity (expected)	0.32	0.36	0.004-0.5
Number of SNPs with a significant HWE deviation = 263 (4.5%)^§^			

^*^Successful SNPs are those with a call frequency > 0.85 and MAF > 0 based on an analysis of 260 trees.

^†^ Successful calls are those with a GenCall score ≥ 0.15 [19].

^§^ Tested using an exact test of HWE and a probability level of 0.9 × 10^-5^ (i.e., Bonferroni-corrected P-value of 0.05 based on 5847 SNPs).

Using logistic regression, we identified eight bioinformatic characteristics significantly related to the ability to distinguish the 5847 successful SNPs from the remaining 2220 SNPs that were assayed (P < 0.05). The order in which the variables entered the model reflects their relative importance: (1) number of datasets in which the SNP was detected, (2) mean number of reads across datasets, (3) number of contigs per isotig, (4) minimum SNP probability across datasets, (5) isotig length, (6) isotig type (i.e., single isotig/isogroup, longest of multiple isotigs/isogroup, or one of multiple isotigs/isogroup), (7) mean SNP probability across datasets, and (8) confidence group (C1-C7). The four variables that did not enter the model were the mean minor allele frequency across datasets, number of isotigs per isogroup, SNP IUPAC code, and Illumina design score.

## Discussion

We developed a reference transcriptome and large SNP database for Douglas-fir that will serve as a resource for a variety of research and breeding applications. We detected SNPs by aligning 454 and Illumina short-read sequences to a reference transcriptome, and then identifying SNP and indel polymorphisms. During this process, we incorporated steps specifically designed to sequence transcripts from diverse genotypes, tissues, and environmental conditions; identify highly-likely SNP positions; and maximize the number of SNPs that can be reliably assayed using an Illumina Infinium II SNP array. A thorough evaluation of the reference transcriptome provides information on the sequences from which the SNPs were derived, including annotations. A set of 278,979 SNPs were deposited in the dbSNP database with submitted SNP ID numbers (ss#) ranging from 523,746,501 to 524,245,331.

### Assembly of the reference transcriptome

We used Newbler v2.3 (Roche GS *De Novo* Assembler) to assemble a reference transcriptome from 454 and Sanger sequences. As for most other non-model organisms [20], we chose pyrosequencing because longer reads are better for *de novo* assembly, and during the sequencing phase of the project, 454 read lengths offered a clear advantage [21]. Sequences were removed from the input dataset by first filtering short and low-quality reads, and reads closely related to adaptor, vector, chloroplast, mitochondrial, rRNA, or retrotransposon sequences (Table 1). For all classes of sequences, the normalized 454 dataset (MG2_*454*_) had smaller numbers of filtered sequences than did the non-normalized dataset (SG_*454*_). Across all datasets (Sanger and 454), rRNA-like sequences represented the largest percentages of filtered reads (3.18%), with retrotransposon-like sequences being second (1.11%). We used procedures similar to those described by Parchman et al. [22] to identify the retrotransposon sequences at the read-level. In lodgepole pine, Parchman et al. [22] found that 3.9% of their normalized 454 reads had characteristics of retrotransposons, but our values were 1.76% and 0.65% for the non-normalized and normalized datasets, respectively (Table 1). Even lower numbers of transposon-like sequences (0.0001 to 0.07% of reads) were reported for other conifer EST datasets [22,23]. Although these sequences could represent transcriptionally active retrotransposons, genomic contaminants are also likely to occur in the cDNA library, particularly when random primers are used for cDNA synthesis [16].

Newbler produces three kinds of output: unique gene models (isogroups), presumed transcript variants (isotigs), and singletons. Prior to the final assembly, we conducted preliminary assemblies using combinations of Newbler parameters, and evaluated the results based on the number of isogroups represented by a single isotig (I1 subset), number of isogroups represented by multiple isotigs (IM subset), and total number of isogroups. We found subtle changes in the resulting assemblies, and ultimately decided to use a minimum overlap length of 45 nt, alignment difference score of −6, and a minimum overlap identity of 96%. For SNP detection, we concluded that these parameters would result in an assembly that balances the detection of false positive SNPs among gene family members that are treated as a single locus versus false negative SNPs that are missed because alleles are treated as separate loci.

We used isotigs and singletons derived from preliminary assemblies to identify and filter reads believed to result from contamination by fungi and bacteria, and to compare levels of contamination between the two 454 datasets. The single-genotype (SG_*454*_) dataset was derived from tissues harvested from the aerial portion of the plant, whereas the multi-genotype (MG2_*454*_) dataset also included washed roots. These analyses suggest that bacterial and fungal contamination was not a serious problem in either dataset (Figure 2), but the true number of contaminating reads is unknown because 26% of the isogroups and 75% of the singletons remained unannotated (Table 5).

In the multi-genotype dataset (MG2_*454*_), the most highly represented bacterial and fungal sequences seemed to be associated with the roots included in this sample. For example, species of *Pseudomonas* are common in soils, where they are associated with plant disease and plant growth promotion [24]. Furthermore, there is a close association between *Pseudomonas fluorescens* and the symbiotic *Laccaria* ectomycorrhizae that infect Douglas-fir roots [25]. Other sequences that were common in the multi-genotype dataset included those related to *Botrytis cinera* (teleomorph *Botryotinia fuckeliana*), a soil fungus that causes grey mold disease in Douglas-fir seedlings [26], *Fusarium circinatum* (teleomorph *Gibberella circinata*), which can cause pitch canker disease on Douglas-fir [27], and *Sclerotinia*, which is interesting because this plant pathogen has never been reported as a pathogen of Douglas-fir (G. Newcombe, personal communication). None of these sequences were common in the single-genotype dataset (SG_*454*_), which did not include roots. Instead, the most highly represented sequences in the single-genotype dataset (*Phanerochaete* and *Antrodia*) belong to genera that include wood-rot fungi which may have been associated with the cambial tissues that were specifically included in this sample. Nonetheless, this sample did contain some reads that are related to *Inonotus*, which is primarily considered a root pathogen (G. Newcombe, personal communication).

### Reference transcriptome

Our first major objective was to assemble a reference transcriptome which could then be used to map reads and identify SNPs in both varieties of Douglas-fir. Our reference transcriptome consists of 25,002 isogroups (unigenes), 38,589 isotigs (transcript variants), 102,623 singletons, and more than 2.5 million 454 reads (Table 2).

Of our 25,002 isogroups, 18,744 are represented by a single isotig (transcript variant) and are inferred to correspond to a single transcript. The remaining 6228 isogroups are represented by multiple isotigs, which suggests they represent alternatively spliced transcripts from the same gene. The mean length of isotigs was 1390 nt and the N50 was 1883. This N50 indicates that 50% of the assembled nucleotides occur in isotigs that are shorter than 1883 nt. These isotigs are about as long as those derived from other recent assemblies of tree transcriptomes. For example, Lorenz et al. [28], assembled 454 reads from 12 conifers using three assemblers. Based on assemblies of 0.4 to 4.1 million reads (depending on species), the average number of contigs (or isotigs) was 54,721, 56,955, and 20,598 using the MiraEST, NGen, and Newbler assemblers, with mean contig lengths of 787, 797, and 1198 nt. Newbler consistently yielded many fewer and longer contigs than did MiraEst and NGen. Using Newbler, the largest dataset of 4.1 million reads (loblolly pine), yielded 48,751 isotigs with a mean length of 1666 nt. In lodgepole pine, NGen was used to assemble a transcriptome from 0.6 million 454 reads, yielding 63,687 contigs with a mean length of 500 nt [22]. Not surprisingly, earlier *de novo* assemblies of transcriptomes of other non-model plants generally used fewer and shorter 454 reads, yielding fewer and shorter contigs [23,29-34].

### Comparison to white spruce

The number of genes in Douglas-fir is unknown, but white spruce, another conifer in the Pinaceae, is estimated to have as many as 32,720 transcribed genes covering as much as 47.3 Mb [16]. This estimate, which is based on Sanger sequencing (272,172 ESTs from cDNA clones), next-generation transcriptome sequencing (7.4 Mb GS-FLX and 59.5 Mb of Illumina GA-II), and genomic sequencing (1.7 Gb GS-FLX), provides a good basis on which to judge the extent of our reference transcriptome. Considering only the longest isotig in each isogroup, our assembly covers 36.1 Mb in isogroups. Therefore, assuming that the white spruce estimates are accurate, and the transcriptomes of Douglas-fir and white spruce are about the same size, our isogroups could represent 76% of the genes and total transcriptome length of Douglas-fir. The total length of singletons was another 36.5 Mb, suggesting that only a modest proportion of these sequences represent unique Douglas-fir transcripts (i.e., missing sequences from already identified genes or unsampled genes). Given the estimated size of the white spruce transcriptome, many of these sequences are probably highly redundant with the assembled isotigs or each other, or represent contaminating sequences from genomic DNA or other organisms.

We compared our isotigs to a white spruce gene catalog of 27,720 unigenes assembled from the 272,172 Sanger sequences described above [16]. Each Douglas-fir isotig was classified into one of seven classes designed to reflect the relative likelihood that reads were assembled correctly into a single locus (Table 3). For example, isotigs having one-to-one matches with white spruce unigenes (E-value < 10^-5^) were classified into the ‘Highest’ confidence class (C1), and isotigs that matched multiple white spruce isotigs and other Douglas-fir isotigs were classified into the ‘Lowest’ class (C6). Isotigs that matched no white spruce unigene were classified into the ‘Unknown’ confidence class (C7).

For the I1 isotigs (1 isotig/isogroup subset), the two largest classes were the ‘Unknown’ and the ‘Highest’ confidence classes, each of which contained ~28% of the 18,774 I1 isotigs. For the IM isotigs (multiple isotigs/isogroup subset), the largest classes were the ‘Lowest’ and the ‘Medium’ confidence classes, each of which contained ~35% of the 19,815 IM isotigs. Overall, these rankings reflect our assumption that overlapping isotigs might be more common among sequences that are incorrectly assembled. These confidence classes were used to prioritize SNPs for the genotyping array, and could also be used to prioritize isotigs for other uses. We subsequently conducted an identical analysis using 35,550 loblolly pine contigs as the reference, and found nearly the same distribution of isotigs among the confidence classes. Across both analyses, we found a total of 6651 no-hit isotigs—that is, isotigs that did not match any spruce or pine contig. This compares to a total of 9960 no-hit isotigs for the spruce analysis, and 8293 no-hit isotigs for pine. These 6651 isotigs deserve attention because they probably represent unique Douglas-fir genes or mis-assembled sequences.

### Annotations

Our second major objective was to annotate the reference transcriptome. We did this by comparing the isotigs and singletons to the Uniref50 and TAIR10 protein databases at an E-value of 10^-5^ (Table 4). For the I1 isotigs, TAIR10 and Uniref50 matches were found for 73.2% and 80.2% of the isotigs, respectively. The percentages of matches for the IM isotigs were considerably lower (52.3% and 55.3%), mostly because we only counted matches when the best hit was identical for all isotigs in an isogroup. Together, these analyses yielded matches for 17,009 (TAIR10) to 18,500 (Uniref50) isogroups. The matches for the singletons were much lower (15.5% and 25.1%). This is expected because these sequences are much shorter and may contain a higher proportion of sequences derived from untranslated transcript regions (e.g., 5’ UTR, 3’ UTR, or unspliced introns) or contaminating genomic DNA. Based on the Uniref50 analyses, most of the isogroup matches had best-hits to plant proteins (Table 5). The modest number of isogroups with hits to conifers (5161) compared to other plants (11,760) probably reflects the much smaller number of available conifer sequences. Among the matched sequences, only 1.33% of the isogroups and 5.18% of the singletons had best hits corresponding to fungal, bacterial, or viral proteins. These could represent contaminating sequences that were not filtered prior to transcriptome assembly.

Because the functions of some of the sequences in the UniRef50 and TAIR10 databases are unknown, we also used the Annot8r annotation tool to identify Douglas-fir sequences that could be assigned a putative function. Specifically, we used Annot8r to query only those sequences in the EMBL UniProt database that are tagged with GO (Gene Ontology) annotations [35]. These analyses found that 14,595 isogroups could be assigned a putative function (GO term; Table 4). If we assume that Douglas-fir has about the same number of genes as white spruce (discussed above), we have putative functional annotations for almost half of the Douglas-fir genes (14,595/32,720 = 44.6%). The GO-annotations were distributed across a wide range of GO slim categories, with no substantial differences among the different categories of isotigs or singletons (Figure 3). Compared to Douglas-fir, many more *Arabidopsis* sequences fell into the “Unknown cellular components” and “Unknown molecular functions” classes, suggesting that these GO slim classes contain *Arabidopsis* genes that are more likely to be absent from Douglas-fir or highly diverged (i.e., resulting in no GO slim assignment for Douglas-fir). Overall, our annotation results suggest that our reference transcriptome (and corresponding SNPs) represent a broad array of genes covering a substantial proportion of the Douglas-fir transcriptome.

### SNP success

Our final two objectives were to identify potential SNPs, and then test a subset of these using an Infinium genotyping array. Across both varieties of Douglas-fir, we identified 278,979 potential SNPs distributed across 20,663 isogroups. We submitted 8769 of these SNPs to Illumina for construction of an Infinium genotyping array. Because bead types are normally lost during the manufacturing process, it was only possible to assay 8067 SNPs (92.0%) on the completed array (Table 7). Based on results from 260 Douglas-fir trees, we identified 5847 reliably scored polymorphic markers, resulting in a conversion rate of 66.7% based on the SNPs submitted to Illumina, and 72.5% based on the number of successful SNP assays (i.e., successful bead types). Using slightly more liberal criteria (i.e., a call frequency of 55% rather than 85%), Eckert et al. [36] reported an overall Infinium conversion rate of ~55% in loblolly pine using a combination of Polyphred, PolyBayes, and a machine learning approach to detect SNPs from Sanger resequencing data. However, using their best SNP detection approach (machine learning), the conversion rate was 66.5%, which is the same as for our submitted SNPs, but lower than the conversion rate for the SNPs we actually assayed (Table 7). These conversion rates are comparable to those reported for other tree species using the Illumina GoldenGate genotyping platform, which ranged from 60.0% to 77.1% in white spruce, black spruce, loblolly pine, and apple [37-39]. In Douglas-fir, the conversion rate for a 384-SNP GoldenGate array was 59% [15]. However, higher conversion rates were reported in sunflower using the Infinium platform [74.9%; [40], and in barley, soybean, wheat, and maize, using the GoldenGate platform [~80-95%; [41-45]. Compared to trees and other outcrossing species, inbred crops may have higher conversion rates because of lower genetic diversity [38,46], resulting in fewer assay failures caused by variation in the primer target sequences.

**Figure 4:**
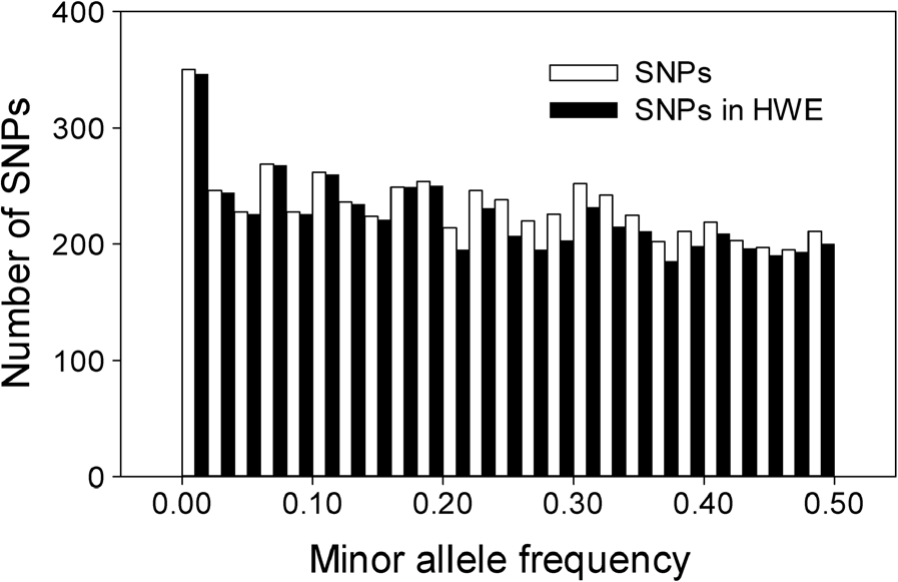
**Distributions of minor allele frequencies for successful Douglas-fir SNPs.** Open bars represent all 5847 successful SNPs. Solid bars represent 5584 successful SNPs that were in Hardy-Weinberg Equilibrium (HWE). Successful SNPs had call frequencies > 0.85 and were polymorphic. Successful calls are those with GenCall scores ≥ 0.15 [19].

**Figure 5:**
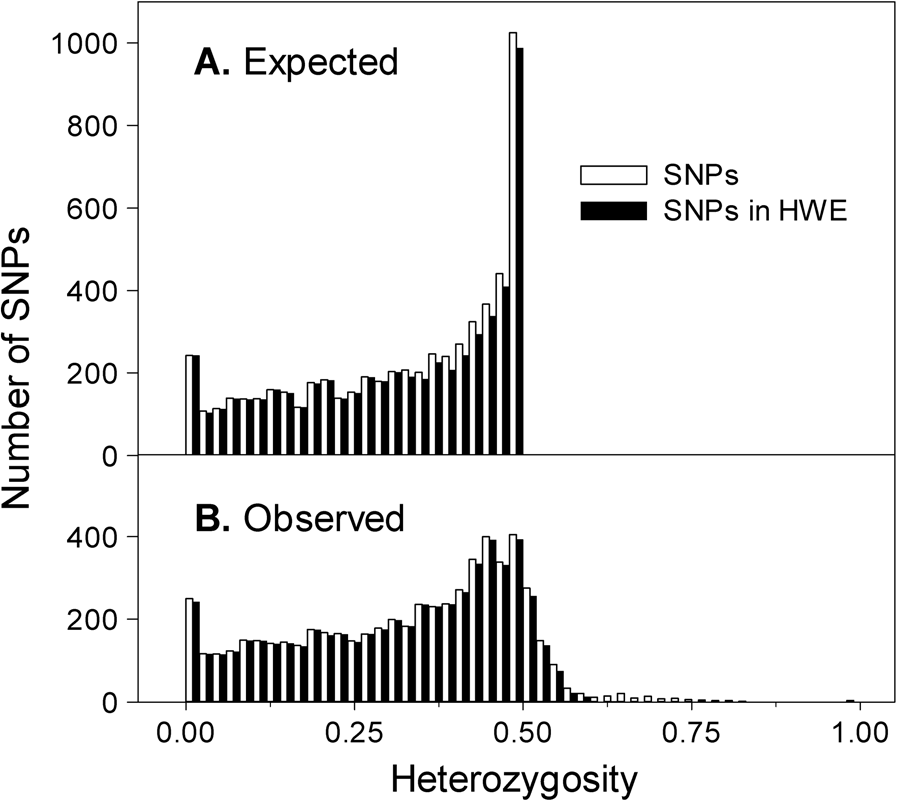
**Distributions of expected and observed heterozygosities for successful Douglas-fir SNPs.** Open bars represent all 5847 successful SNPs. Solid bars represent 5584 SNPs that were in Hardy-Weinberg Equilibrium (HWE). Successful SNPs had call frequencies > 0.85 and were polymorphic. Successful calls are those with GenCall scores ≥ 0.15 [19].

Our 5847 successful SNPs had a median GC50 score of 0.87 and a median call frequency of 1.00 (Table 8). Because we filtered SNPs based on SNP probabilities and other metrics that are positively associated with MAF, our successful SNPs had high MAFs (median = 0.24) and heterozygosities (median = 0.36). Therefore, their polymorphic information content is probably much higher than that of randomly selected SNPs. Selection of SNPs with high MAFs also resulted in a very flat frequency distribution (MAF range = 0.002-0.500; Figure 4) and a moderately flat distribution for observed heterozygosity (Figure 5).

We also identified 263 SNPs (4.5%) that deviated significantly from HWE based on a Bonferroni-corrected P-value of 0.05. In general, HWE deviations may result from genotyping errors, non-random mating, selection, mutation, gene flow/admixture, or relatedness among samples. However, for the SNPs with observed heterozygosities much greater than 0.5 (Figure 5), we may also be detecting polymorphisms among nearly identical paralogs [47]. Although deviations from HWE are often used to filter SNPs used in association studies, no consensus has emerged on the appropriate P-value to use [48]. However, probabilities of 10^-5^ to 10^-6^ are typically used to filter SNPs in genome-wide association studies [49]. We used the Bonferroni correction because this approach was previously used to filter SNPs in association studies of Douglas-fir and loblolly pine [15,36], and because the unadjusted threshold of 0.9 × 10^-5^ is consistent with other common practices [49]. If these 263 SNPs are not used in association genetic studies or other analyses, the number of non-filtered SNPs would be reduced to 5584. In loblolly pine, 1.46% (45/3082) SNPs deviated significantly from HWE using the Infinium platform [36].

We subsequently used logistic regression to test whether successful SNPs could be predicted from bioinformatic characteristics. Although eight variables entered the prediction model, the model had little predictive power. This is not surprising because most of the assayed SNPs were highly selected based on these same variables, so the independent variables had little variation. Compared to random selection from our pool of 8067 SNPs, the prediction model only increased the probability of selecting successful SNPs from 72.5% to 73.8%. These results suggest that we could have relaxed our SNP selection criteria with little effect on SNP success―i.e., many more successful SNPs would have been identified had we developed and tested a much larger genotyping array.

We also used multiple linear regression to determine whether any of the five SNP characteristics listed in Table 8 (i.e., excluding expected heterozygosity) could be predicted from the across-dataset variables used for SNP filtering. These predictor variables included 12 continuous and categorical variables reflecting SNP frequencies, SNP probabilities, numbers of covering reads, SNP repeatabilities across datasets, Illumina assay design scores, isotig characteristics, types of SNP (i.e., IUPAC codes), and SNP confidence classes. Although all models were highly significant, the R^2^ values were all below 4%, except for MAF and observed heterozygosity. For MAF, ~20% of the variation was explained by three variables—mean SNP frequency, mean SNP probability, and isotig length. For observed heterozygosity, ~16% of the variation was explained by these three variables plus the mean number of covering reads.

### A SNP resource for genomic selection

One of our key long-term goals is to test whether genomic selection can be used to enhance Douglas-fir breeding. Genomic selection, or whole genome selection, is a type of marker-assisted selection that uses dense marker coverage to track alleles for most or all quantitative trait loci (QTL) in the genome [6]. If very large numbers of markers are used, most or all QTL will be in linkage disequilibrium with at least one marker, particularly in small populations. Genomic selection involves two steps [50]. First, a genomic prediction model is developed using phenotypes and marker genotypes measured on a test or ‘training’ population. Second, individuals are selected from a related population of selection candidates based on breeding values predicted from the marker genotypes alone.

The number of markers needed for accurate genomic selection varies widely, depending on the genome length (cM), effective size of the breeding population (*N*_e_), number of QTLs, heritability, number of generations without model retraining, and other factors [50-52]. For example, a 50K SNP chip has been used by dairy cattle breeders since 2008, and a 777K SNP chip is now available that may be useful for making selections across-breeds [53]. In contrast, it may be possible to use many fewer markers in forest trees because small breeding populations can be used to increase linkage disequilibrium (LD) [51]. In a simulation study of genomic selection in forest trees, ~2 markers per cM were sufficient to achieve the same accuracy as BLUP-based phenotypic selection when *N*_e_ was ≤ 30, but as many as 20 markers per cM might be needed for an *N*_e_ of 100 [51]. Iwata et al. [54] came to a similar conclusion in a simulation study of a generic conifer breeding program. They concluded that efficient genomic selection would be achieved in a small breeding population (*N*_e_ = 25) using one marker per cM, and that accuracies could be increased by using greater marker densities. Assuming a genome length of ~2000 cM for Douglas-fir [55], these values (i.e., 1–20 markers per cM) are equivalent to about 2,000 to 40,000 SNPs. Empirical results support the results of these simulation studies. In two small populations of *Eucalyptus* (*N*_e_ = 11 and 51), the accuracy of genomic selection equaled that of BLUP-based phenotypic selection using > 3000 DArT markers [56]. Similar results were also observed in a loblolly pine population (*N*_e_ ~40) using 4825 SNP markers [57].

What is the size of our SNP resource? If we multiply the number of potential SNPs by our SNP conversion rate (72.5%; Table 7), we obtain an estimate of 202,260 true SNPs. However, if we had tested all 278,979 SNPs on the genotyping array (i.e., by relaxing our selection criteria), the SNP conversion rate may be lower. In contrast, the number of potential SNPs would have been much larger had we used a SNP probability threshold of 10^-3^ (337,938 SNPs) or even 10^-2^ (440,550 SNPs), but the SNP conversion rate may have been lower as well. Balancing these factors, a reasonable estimate for the number of true SNPs is ~200,000. Second, what is the number of SNPs that can be genotyped using an Infinium II assay? This can be judged by the number of acceptable design scores. For example, using a SNP probability of 10^-4^ (278,979 potential SNPs) and a probability of flanking variants (*P*_F_) of 10^-1^, we obtained 95,478 SNPs with design scores ≥ 0.6. Again, assuming a 72.5% conversion rate, the number of successfully genotyped SNPs is estimated to be 69,221. We also tested the effects of using the same SNP probability (*P*_S_ < 10^-4^), but with lower flanking probabilities (*P*_F_ < 10^-2^, 10^-3^, and 10^-4^; Figure 1). Using a *P*_F_ value of 10^-4^, for example, the number of SNPs with an acceptable design score was 150,025 (Figure 1), and the number of successfully genotyped SNPs is estimated to be 108,768. Although SNP conversion rates may differ among these scenarios, our SNP resource seems more than sufficient to pursue genomic selection in Douglas-fir.

Although we identified 278,979 potential SNPs, they are not distributed uniformly across the genome, which would be optimal for genomic selection. Therefore, it would be better to use the number of isogroups with SNPs (n = 20,663) to judge the effectiveness of our SNP resource for genomic selection. However, the large number of SNPs we detected means that it should be possible to genotype nearly all of these loci. Furthermore, if much of the genetic variance of interest is explained by variants in or near transcribed genes, then these markers may be more efficient than randomly distributed markers. Approaches for increasing the number of loci with SNPs could involve mapping more reads to our reference transcriptome (n = 25,002 isogroups), increasing the coverage of our reference transcriptome (yielding perhaps 30,000 to 40,000 loci), and relying on genomic sequencing to develop additional markers in non-transcribed regions.

## Conclusions

We conclude that our current dataset of 278,979 potential SNPs will translate into as many as ~200,000 true SNPs, and as many as ~69,000 SNPs that could be genotyped at ~20,000 gene loci using an Infinium II array. Furthermore, we already have enough validated SNP markers (5847 markers in 5439 isogroups) to conduct realistic tests of genomic selection on small breeding populations of Douglas-fir. Assuming a density of 2.5 markers per cM (5000 SNPs/2000 cM), we should be able to practice effective genomic selection in populations up to ~30 *N*_e_[51]. However, because current breeding populations now average about 220 *N*_e_ (K. Jayawickrama, personal communication), we will either need more markers to practice genomic selection, or genomic selection will need to focus on smaller populations (e.g., sublines). Ultimately, our reference transcriptome and SNP resource will enhance Douglas-fir breeding and allow us to better understand landscape-scale patterns of genetic variation and potential responses to climate change.

## Methods

### Plant materials and RNA preparation

#### Reference transcriptome

We used three sets of coastal Douglas-fir sequences to construct the reference transcriptome (Table 1). In this section, we describe the plant materials and general sequencing strategies used for each dataset. Detailed laboratory methods are described subsequently.

The goal of the first multi-genotype dataset (MG1_*SANG*_) was to include existing Sanger sequences from a diverse set of genotypes known to be expressing functionally important genes. The MG1_*SANG*_ dataset was prepared by combining Sanger sequences derived from three ‘cold hardiness’ cDNA libraries (CA, MH, and CD) and one ‘actively growing’ (GR) library [58]. Seedlings used for the cold acclimating (CA) library were collected in September, October, and November; seedlings used for the maximum hardiness (MH) library were collected in December and January; and seedlings used for the cold deacclimating (CD) library were collected in February, March (2 dates), and April in Corvallis, OR. On each date, 10 seedlings were collected from a single orchard seedlot, and total RNA was extracted separately from needles, stems, and buds. The parents of these seedlings originated from a low-elevation population near Toledo, OR. Total RNA was isolated at Oregon State University (OSU) according to Chang et al. [59], except that the RNA was subsequently purified on RNeasy columns (QIAGEN, Valencia, CA, USA). Equal amounts of total RNA were pooled from each tissue prior to sequencing. Sanger sequences from the cold hardiness libraries (CA = 3,949; MH = 3,701; and CD = 3,684 sequences) were combined with 6,760 sequences from the GR library prepared from actively growing seedlings harvested from the greenhouse during their first growing season [55].

The goal of the second multi-genotype dataset was to increase the number genotypes, tissues, and physiological conditions, while also increasing sequence depth and coverage by using 454 pyrosequencing. The resulting MG2_*454*_ dataset consisted of Roche 454 sequences derived from three tissue collection regimes. First, on each of five dates between September and April, we harvested 6 or 12 first-year seedlings and separated them into needles, stems, and buds. These seedlings were grown outdoors in Corvallis, OR, but their seed orchard parents originated from a low-elevation population near Coos Bay (CB), OR [58]. Second, we harvested three seedling tissues on five dates between July and January from a total of 79 seedlots provided by the Cottage Grove Nursery of Plum Creek Timber Company. On all five dates, we harvested buds (i.e., elongating apices or resting buds), shoots (stems plus needles), and roots. On two of the dates when the seedlings were large enough, we also harvested lower stems without needles. These seedlings were grown outdoors in Corvallis, and some were subjected to water stress to induce the expression of genes associated with adaptation to cold and drought. Third, we collected elongating shoots from ramets of two clonal genotypes growing at the Lebanon Forest Regeneration Center managed by Roseburg Forest Products. Because these shoots were collected during June from trees that had been stimulated to produce reproductive buds, they are expected to contain differentiating male and female flowers (strobili). Total RNA was isolated at OSU as described above, or at the University of Georgia (UGA) as described by Lorenz et al. [60]. Individual RNA samples were pooled in an attempt to have mRNAs from buds, stems, needles, and roots equally represented in the resulting cDNA libraries.

The goal of the single-genotype dataset was to expand our representation of genes from mature trees and increase sequence depth and coverage by using 454 pyrosequencing. The resulting SG_*454*_ dataset consisted of Roche 454 sequences derived from five tissues collected on 8 July from two mature ramets of a single clonal genotype growing at the Lebanon Forest Regeneration Center. We collected terminal shoots, stems, and resting buds (all from current year branches), plus cambial tissue and developing seeds from immature cones. Total RNA was isolated at UGA as described by Lorenz et al. [60], and 36 to 130 μg of total RNA was pooled from each tissue prior to cDNA synthesis.

#### Illumina short-read sequences

The goal of the Illumina sequencing was to enhance SNP detection by increasing the number and genetic diversity of the sequences to be mapped to the reference transcriptome. We used coastal and interior Douglas-fir to produce four sets of Illumina short-read sequences. One set of coastal Douglas-fir sequences (MG2_*IL*_) was derived from the same pooled RNA sample that was used to construct the MG2_*454*_ dataset described above. The Coos Bay (CB_*IL*_) dataset was derived from a subset of the Coos Bay RNA samples described above, plus replicate samples harvested on some of the same dates. We used six bud samples and two needle samples for a total of eight Illumina sequencing runs. The Yakima (YK_*IL*_) dataset was prepared using the same collection protocol and sequencing protocol as for the CB_*IL*_ dataset, but the seedlings were derived from parents growing in a high-elevation inland population near Yakima, Washington that is thought to represent the interior variety of Douglas-fir [61]. Total RNA was isolated at OSU as described above.

The interior Douglas-fir samples (INT_*IL*_ dataset) were collected from mature trees growing in a provenance test near Vernon, B.C., Canada [62] and the Cherrylane Seed Orchard in northern Idaho. Young shoots were collected from the provenance test in early May from two trees from each of 26 seedlots collected from Arizona and New Mexico in the south, to British Columbia and Washington state in the north. Approximately equal amounts of total RNA were pooled from recently flushed buds, stems, young needles, and mature needles. The seed orchard samples were collected in early June from 18 trees originating from northern Idaho. Approximately equal amounts of total RNA were pooled from stems and needles harvested from recently flushed shoots. The two pooled RNA samples were then combined for Illumina sequencing.

### DNA sequencing

#### Reference transcriptome

The MG1_*SANG*_ dataset was produced via Sanger sequencing. For the CA, MH, and CD libraries, pooled samples of total RNA were used by Evrogen JCS (Moscow, Russia) for double-stranded cDNA synthesis using the SMART approach [63], and the cDNAs were normalized using DSN normalization [64]. The resulting cDNAs were directionally inserted into the pAL17.1 vector and transformed into E. coli. SymBio Corporation (Menlo Park, CA) amplified the cDNA clones using rolling circle amplification, and then sequenced about 4,000 cDNA clones per library using a MegaBASE 4000 sequencer (GE Healthcare, Little Chalfont, UK). The non-normalized GR library was prepared and sequenced (Sanger) as described by Krutovsky et al. [55]. Sanger sequences were archived under GenBank accession numbers CN634509-CN641229 and ES417751-ES429084.

The MG2_*454*_ and SG_*454*_ datasets were produced via 454 pyrosequencing. For the MG2_*454*_ dataset, mRNA isolation, cDNA synthesis, and DNA sequencing were performed by the University of Illinois Carver Biotechnology Center using the SuperScript Double-Stranded cDNA Synthesis Kit (Invitrogen, CA) and GS Titanium Library Preparation kit (454 Life Sciences, Branford, CT). The cDNA library was normalized using the Trimmer Direct Kit (Evrogen), and then sequenced using the 454 GS-FLX platform. For the SG_*454*_ dataset, cDNA synthesis was performed by the U.S. Department of Energy Joint Genome Institute (JGI) using the SMART PCR cDNA Synthesis Kit (Clontech, Mountain View, CA). The resulting non-normalized cDNA library was sequenced by JGI using the 454 GS-FLX platform. The raw 454 sequences were deposited in the NCBI Sequence Read Archive (SRA) under accession numbers SRA023776 and SRA051424.

#### Illumina short-read sequences

The CB_*IL*_ and YK_*IL*_ libraries were constructed at the USDA Forest Service’s Pacific Northwest Research Station using Illumina mRNA-Seq Prep Kits (San Diego, CA) with minor modifications. To obtain strand-oriented reads, we used dUTP for second-strand synthesis, and then selectively destroyed the dUTP-containing strand before the PCR-enrichment step as described by Parkhomchuk et al. [65]. Libraries were constructed using multiplex sequencing adapters [66], and single-end reads of 80 nt were obtained using multiplex sequencing on an Illumina Genome Analyzer IIx at the OSU Center for Genome Research and Biocomputing or Harvard FAS Center for Systems Biology. The MG2_*IL*_ and INT_*IL*_ cDNA libraries were constructed using the standard Illumina mRNA-Seq Sample Prep Kit, and then sequenced on an Illumina Genome Analyzer IIx for 2x101 cycles at the Carver Biotechnology Center. The RNA previously used for the MG2_*454*_ dataset was also used for the MG2_*IL*_ library, and the RNA isolated from interior Douglas-fir was used for INT_*IL*_. The raw Illumina sequences were deposited in the NCBI SRA under accession number SRA051424.

### Pre-assembly sequence processing for the reference transcriptome

Prior to assembly, sequences in the MG1_*SANG*_, MG2_*454*_, and SG_*454*_ datasets were cleaned as described below (Figure 1). For the MG2_*454*_ and SG_*454*_ datasets, we used Roche’s sffinfo utility to trim primer and adaptor sequences and produce raw FASTA and quality files from the SFF files. For Sanger sequences (MG1_*SANG*_), we performed these same functions using phred [67]. We then used the SnoWhite pipeline (http://www.evopipes.net/snowhite.html), which combines Seqclean and TagDust, to remove or mask polyA/T tracts; short, low-quality and low-complexity sequences; and reads matching chloroplast, mitochondrial, rRNA, or retrotransposon sequences. Our filtering database contained (1) vector, adapter, linker, and primer sequences from NCBI’s UniVec database (http://www.ncbi.nlm.nih.gov/VecScreen); (2) chloroplast, mitochondrial, and ribosomal RNA (rRNA) sequences from 21 to 50 species that included conifers, *Arabidopsis*, and *Nicotiana* (GenBank; http://www.ncbi.nlm.nih.gov/genbank); (3) a nearly-complete reference of the coastal Douglas-fir chloroplast genome (GenBank JN854170; [68]); and (4) retrotransposon sequences from 27 species, including *Arabidopsis* and *Oryza* sequences from the Plant Repeat Database (http://plantrepeats.plantbiology.msu.edu/) and conifer sequences obtained from GenBank as described by Parchman et al. [22]. Our final database of non-redundant filter sequences was prepared by processing all filter sequences through the NCBI BLASTclust program [69] with the identity and coverage parameters set to 90% (i.e., pairwise matches require sequences to be 90% identical over 90% of their lengths). We then used the SnoWhite pipeline to filter reads that had ≥ 96% sequence identity to any sequence in this filtering database (Figure 1, Step 3)

We also filtered bacterial- and fungal-like sequences from the datasets used for transcriptome assembly. These sequences were filtered by first conducting a *de novo* assembly of each 454 dataset (MG2_*454*_ and SG_*454*_) using Newbler v2.3 (Figure 1, Step 4; discussed below). The resulting isotigs and singletons (plus the Sanger sequences from the dataset MG1_*SANG*_) were screened for homology using BLASTN against a dataset of 27,720 white spruce unigenes [16]; http://www.arborea.ca]. Non-matches (E-value > 10^-5^, bit score < 50, and identity < 96%) were subsequently screened using BLASTN and two local databases: the NCBI nucleotide collection (nr/nt) and NCBI non-human, non-mouse ESTs (est-others). Assembled isotigs and singletons were filtered if the best hit had a bit-score > 50 and an E-value < 10^-10^, and the corresponding genus name was found in a custom database of 162,679 bacterial and 59,139 fungal names downloaded from the NCBI Taxonomy database (http://www.ncbi.nlm.nih.gov/Taxonomy) (Figure 1, Step 5). After we removed the singletons and all reads that assembled into the contaminating isotigs, the original reads were re-assembled as described below (Figure 1, Step 6). We also used the results from these analyses to compare the number of contaminating fungal and bacterial reads between the two 454 datasets.

### Assembly of the reference transcriptome

We used Newbler v2.3 (Roche GS *De Novo* Assembler v2.3; Roche Life Sciences, Inc.) to assemble the reads in the MG1_*SANG*_, MG2_*454*_, and SG_*454*_ datasets into a single reference transcriptome consisting of isogroups (unigene models), isotigs (presumed transcript variants), and singletons ≥ 100 nt (Figure 1, Step 6). Prior to the final assembly of all datasets, we first evaluated the impact of alternative assembly parameters. *De novo* assemblies were run using the transcriptome (-cdna) option, minimum read length (-minlen) of 40 nt, isotig length threshold (-icl) of 40 nt, a large contig threshold (-l) of 100 nt, plus a factorial arrangement of the following parameters: minimum overlap lengths of 35 and 45 nt; alignment difference scores of -2 and -6; and minimum overlap identities of 82% to 98%. The 20 resulting assemblies were evaluated based on the total numbers of isogroups, number of isogroups represented by a single isotig (I1 subset), and the number of isogroups represented by multiple isotigs (IM subset). We also evaluated the assemblies by comparing the assembled isogroups to white spruce unigenes using the approach described below. Based on these evaluations, we performed the final Newbler assembly using a minimum overlap length of 45 nt, alignment difference score of -6, and a minimum overlap identity of 96%. We clustered the resulting isotigs using Vmatch (http://www.vmatch.de; –dbcluster p_*small*_ = 99 and p_*large*_ = 99) to form a non-redundant set of sequences, and then calculated the assembly statistics shown in Table 2 using custom Perl scripts. The reference transcriptome (i.e., 37,177 isotigs ≥ 200 nt) has been deposited at DDBJ/EMBL/GenBank under accession GAEK01000000.

### Comparison to white spruce and loblolly pine

We compared the Douglas-fir assembly to a set of white spruce unigenes using SCARF, a sequence assembly tool designed for assembling 454 EST sequences against a reference sequence from a related species (Figure 1, Step 8) [17]. We downloaded 27,720 white spruce unigenes constructed from Sanger sequenced ESTs [16]; http://www.arborea.ca], and then used SCARF to determine where Douglas-fir isotigs matched white spruce unigenes. The combination of MegaBLAST parameters [70] used in the SCARF analysis resulted in matches with a minimum length of 40 nt, minimum identity of 77%, minimum bitscore of 80, and maximum E-value of 2 × 10^-13^. Using this information, we defined seven types of structural relationships (C1 to C7) between Douglas-fir isotigs and white spruce unigenes, and then assigned the isotigs to these classes based on three criteria (Table 3). First, we classified each isotig according to the number of white spruce matches: no match (C7), one match (C1, C3, C4), or matches to multiple white spruce unigenes (C2, C5, C6). Multiple matches were counted only when the percent identities were within 5% of the best match. Second, we determined whether other isotigs matched the same white spruce unigene, resulting in isotigs that were classified as having no matching partners (C1, C2), and those that did (C3 to C6). Finally, for the isotigs with matching partners, we determined whether the partners overlapped each other (C4, C6) or not (C3, C5). We then assigned relative confidence scores to each isotig assembly based on these relationship classes: C1 = Highest; C2 and C3 = Higher; C4 and C5 = Medium, C6 = Lower; and C7 = Unknown.

We conducted the same SCARF analysis using loblolly pine as the reference, but these analyses were completed after the SNP array was constructed and tested. These analyses were conducted using 35,550 contigs that comprise the first release of the PineDB transcriptome assembly (PineDB v1.0; June 15, 2012; http://bioinfolab.muohio.edu/txid3352v1/interface/download.php).

### Annotation

We annotated the isogroups using a local tBLASTX [69] search against the Uniref50 release 2010_09; [71] and TAIR10 (TAIR10_pep_20101214; [72]) databases using an E-value of 10^-5^ (Figure 1, Step 8). We then summarized the results separately for the I1 isogroups, IM isogroups, and singletons. For the IM set of isogroups, a hit was counted only if all isotigs matched the same protein in the database; otherwise this isogroup was considered unannotated. We also annotated sequences using the Annot8r pipeline (http://www.nematodes.org/bioinformatics/annot8r/index.shtml), which assigns GO terms [73], EC numbers (http://www.chem.qmul.ac.uk/iubmb/), and KEGG pathways [74] to protein or nucleotide sequences from non-model organisms based on sequence similarity to protein sequences in the EMBL UniProt database (http://www.uniprot.org/). We also assigned GO-slim terms to the isogroups and singletons using the results from the TAIR10 tBLASTX search. We extracted GO-slim terms for the matching *Arabidopsis* accessions from the TAIR10 database, and then compared the distributions of GO-slim terms for the I1 isogroups, IM isogroups, and singletons versus the distribution of GO-slim terms for all 35,386 *Arabidopsis* accessions in the TAIR10 database (http://ftp.arabidopsis.org/home/tair/Ontologies/Gene_Ontology/). Finally, we assigned taxonomic affiliations to the isogroups and singletons using the results from the UniRef50 tBLASTX search described above. We extracted the taxonomic assignment for each best-hit, and then summarized them according to the categories shown in Table 5.

### Processing of Illumina short-read sequences and analysis of sequence orientation

Illumina short read sequences were mapped to the transcriptome reference to identify SNPs. Some of the short-read sequences contained strings of nucleotides with a quality score of 2 (i.e., ‘B’ ascii character), which Illumina uses to indicate that these calls should not be used for downstream analysis. Therefore, we changed these positions to ‘N’s before read mapping and SNP detection (Figure 1, Step 7).

We used the strand-oriented reads from the CB_*IL*_ and YK_*IL*_ libraries to infer the orientation of the isotigs and singletons. We used Bowtie v 0.12.7 (−M 1, -q, –n 2; [75]) and custom R scripts to count the number of unique alignment locations where reads were mapped as direct Illumina output (D) and as their reverse complements (C). For each isotig and singleton, we summed D and C across both strand-specific datasets, and then used a two-tailed binomial test to test whether C was significantly greater or less than D (P < 0.05), which would indicate the corresponding isotig or singleton is in the forward (+) or reverse (−) orientation, respectively.

### SNP detection

#### Flanking variants

The first step toward identifying likely SNPs and designing SNP assays was to identify flanking variants (SNPs and indels) using permissive criteria (Figure 1, Step 9). We combined the Sanger and 454 sequences (MG1_*SANG*_, MG2_*454*_, and SG_*454*_) into a single dataset, and then aligned them to the reference using the BWA-SW program with default parameters [76]. For the Illumina sequences (CB_*IL*_, YK_*IL*_, and INT_*IL*_), we used the Novoalign short-read aligner with default parameters (Novocraft Technologies; http://www.novocraft.com). We used SAMTools [77] to output the alignment results to the BAM format, and then used mpileup, BCFTools, VCFutils, custom Perl scripts, and SAS (Statistical Analysis System, Cary, NC) to extract and summarize sequence variants. For these programs, we used the following parameters: 20,000 = maximum number of reads for calling a SNP, 20 = minimum mapping quality, and 20 = minimum base quality to identify putative SNPs. These SNPs were subsequently filtered using more stringent criteria. Although we recorded indels found within the query dataset or between the query dataset and the reference, we only recorded SNPs found within the queried dataset. That is, if the query dataset differed from the reference, but had no called SNP itself, we treated the variant as a sequencing error. Because our input sequences were derived from pooled samples, we did not filter variants based on probability values from BCFTools (i.e., we used -p = 2.0 for the BCFTools view and VCFutils programs). Instead, we estimated SNP and indel probabilities from the mpileup output using a custom Perl script that implemented the methods described by Wei et al. [78], using a MAF value of 0.01 and sequence error rate of 0.01. We also used this Perl script to remove variants that had a total read depth < 5 or < 2 alternative alleles in the dataset. For each dataset, we compared variants detected using the BCFTools/VCFutils programs versus our custom Perl script, removed indels from the 454 dataset, merged the five datasets, and then removed other variants that did not meet a flanking probability threshold (*P*_F_) of 0.10 in any single dataset or pooled across datasets (Figure 1, Step 9). The pooled across-dataset probability was calculated using a chi-square test with 10 degrees of freedom, where *X*^2^ equals −2Σln(*p*_i_), and *P*_*i*_ is the SNP probability for each of the five datasets [79]. We then used a Perl script to generate a reference sequence for each dataset that identified all retained indel and SNP positions using IUPAC codes, and these were combined to create a comparable sequence for Douglas-fir.

#### Target SNPs

We filtered flanking SNPs to obtain sets of ‘target SNPs’ that could serve as a resource for future genotyping assays. In Figure 1, we show three output datasets based on target SNP probabilities (*P*_S_) of 10^-2^, 10^-3^, and 10^-4^ (Figure 1, Step 10). To avoid redundant SNPs, this database was developed using only the longest isotig from each isogroup. For these datasets, we retained bi-allelic SNPs that were not near a high-quality indel (i.e., did not receive a BCFTools code of “G” in any dataset), had a mapping quality score > 40 in at least one dataset, and probabilities < 10^-2^, 10^-3^, or 10^-4^ in at least one dataset. Using a SNP probability of 10^-4^, these criteria resulted in 278,979 potential SNPs for which we obtained Infinium design scores. Design scores were obtained using four different sequence datasets constructed using flanking probabilities (*P*_F_) of 10^-1^, 10^-2^, 10^-3^, and 10^-4^ (Figure 1, Step 11). The dataset of 278,979 potential SNPs constructed using a *P*_S_ of 10^-4^ and *P*_F_ of 10^-1^ was used as the starting point for constructing a genotyping array. These SNPs have been deposited in the NCBI dbSNP database under submitter handle HOWE_OSU, with ss numbers ranging from 523,746,501 to 524,245,331.

#### Infinium genotyping array

We used additional criteria to filter the target SNPs to obtain 8769 SNPs for testing on an Infinium II genotyping array (Figure 1, Step 12). During this filtering step, we did not consider SNPs from isotigs having low confidence scores (C5 or C6). First, we selected SNPs in genes that were differentially expressed during cold acclimation [58]; unpublished data] or had annotations suggesting they were associated with growth, phenological traits, stress resistance, or adaption to temperature or drought. For these SNPs, we selected as many as two SNPs per isotig, excluding SNPs within 50 nt of each other. For the remaining SNPs, we removed those not found in at least two datasets, and then retained the most probable SNP in each isotig (i.e., based on the mean probability across all datasets). In the final filtering step, we retained all SNPs in differentially expressed genes (see above), and then filtered the remaining SNPs if they required two probes to assay (i.e., Infinium I assay type = A/T and C/G SNPs), or had a design score < 0.60, fewer than 10 quality reads, or a frequency < 0.05 (i.e., based on mean values across datasets). These criteria, which yielded 8769 SNPs, were specifically chosen to be compatible with an Infinium II array [18] that has a capacity of 9,000 attempted bead types.

We tested the Infinium array by genotyping 260 trees of coastal Douglas-fir. DNA was isolated from ~50 mg of frozen needles using the DNeasy Plant 96 Kits (QIAGEN), genotyping was performed by the UC Davis Genome Center according to protocols from Illumina, and the resulting data were analyzed using Illumina GenomeStudio software v2011.1 [80].

We assessed the quality of the resulting SNP loci based on the Illumina GenTrain scores, GenCall scores, SNP call frequencies, MAFs, and probabilities of deviation from HWE (Figure 1, Step 13) [80]. Each of these measures ranges from 0 to 1. GenomeStudio software uses a custom algorithm to cluster the data for each locus into homozygous and heterozygous classes, and the GenTrain score reflects the quality of these clusters. The calling algorithm then uses the GenTrain model and signal intensities to assign (“call”) a genotype for each locus and tree. The GenCall score reflects the quality of this assignment, and can be used to judge the quality of an individual SNP call, a SNP locus, or DNA sample. For example, the median GenCall score (50% GenCall score) is often used to judge the quality of SNP loci. Another measure of locus quality is the call frequency, or call rate, which is the number of successfully called SNP genotypes divided by the number of DNA samples (260 in our case). Based on the recommendation for the Infinium platform [19], we considered calls with GenCall scores < 0.15 as unsuccessful (“no calls”). In this paper, we report the numbers and characteristics of high-quality SNP loci, which we defined as loci that were polymorphic in our sample of 260 trees with call rates ≥ 85%. We also identified SNPs that deviated from HWE using the exact test described by Wigginton et al. [81] and a probability level of 0.9 × 10^-5^ (i.e., Bonferroni-corrected P-value of 0.05 based on 5847 SNPs). Finally, we used SAS Proc Logistic and stepwise model selection to determine whether the high-quality SNPs could be predicted from 12 SNP bioinformatic characteristics.

## Abbreviations

BAM: Binary version of a SAM (Sequence Alignment/Map) file; CBIL: Coos Bay sequence database produced by Illumina sequencing; cM: centiMorgan; EC: Enzyme Commission; EMBL: European Molecular Biology Laboratory; EST: Expressed sequence tag; GO: Gene Ontology; HWE: Hardy-Weinberg equilibrium; INTIL: Interior Douglas-fir sequence database produced by Illumina sequencing; IUPAC: International Union of Pure and Applied Chemistry; KEGG: Kyoto Encyclopedia of Genes and Genomes; MAF: Minor allele frequency; Mb: Megabase; MG1SANG: Coastal Douglas-fir multi-genotype sequence database #1 produced by Sanger sequencing; MG2454: Coastal Douglas-fir multi-genotype sequence database #2 produced by Roche 454 sequencing; NCBI: National Center for Biotechnology Information; nt: Nucleotide; QTL: Quantitative trait locus; SG454: Coastal Douglas-fir single genotype sequence database produced by Roche 454 sequencing; SNP: Single nucleotide polymorphism; SSR: Simple sequence repeat; YKIL: Yakima sequence database produced by Illumina sequencing

## Competing interests

The authors declare that they have no competing interests.

## Authors’ contributions

GTH conceived and coordinated the project, helped with the bioinformatics of transcriptome evaluation and SNP detection, designed the Infinium array, analyzed the Infinium results, and wrote the final manuscript with written contributions from JY and BK. JY had primary responsibility for the design and implementation all aspects of transcriptome assembly and annotation, helped with the bioinformatics of transcriptome evaluation and SNP detection, collected plant tissues, isolated RNA, and helped write the manuscript. BK helped prepare and sequence the CB_*IL*_ and YK_*IL*_ libraries, archived the short-read datasets, provided technical advice, and helped write the manuscript. RC conceived and guided the preparation and sequencing of the CB_*IL*_ and YK_*IL*_ libraries and provided strategic advice. SK helped design the plant materials protocol, maintained and harvested plants, and isolated RNA. PD conducted the bioinformatics and statistical analyses used to orient the transcriptome assembly. WWL isolated RNA and worked with JGI to prepare the SG_*454*_ dataset. JFDD conceived and guided the preparation of the SG_*454*_ dataset. All authors read, edited, and approved the final manuscript.

## Supplementary Material

Additional file 1: Tables S1 and S2 Characteristics of the reference transcriptome isotigs and isogroups. Information on the isotigs and their associated isogroups is found on the ‘Isotig data (S1)’ worksheet and variable descriptions are found on the ‘Variable descriptions (S2)’ worksheet. Click here for file

Additional file 2: Tables S3 and S4 Characteristics of the reference transcriptome singletons. Information on the singletons is found on the ‘Singleton data (S3)’ worksheet and variable descriptions are found on the ‘Variable descriptions (S4)’ worksheet. Click here for file

Additional file 3: Tables S5 and S6 Characteristics of the 278,979 putative SNPs summarized in Table 6. Information on the SNPs and their associated isogroups is found on the ‘Target SNP data (S5)’ worksheet and variable descriptions are found on the ‘Variable descriptions (S6)’ worksheet. Click here for file

Additional file 4: Tables S7 and S8 The locus summary report from Illumina’s GenomeStudio v2011.1 software and additional derived variables are found on the ‘SNP Infinium results (S7)’ worksheet. Descriptions of GenomeStudio variables (modified from, [19,80]) and other variables are found on the ‘Variable descriptions (S8)’ worksheet. Click here for file

## References

[B1] Stenson PD Ball EV Howells K Phillips AD Mort M Cooper DN The Human Gene Mutation Database: Providing a comprehensive central mutation database for molecular diagnostics and personalized genomics Hum Genomics 2009 4 69 72 10.1186/1479-7364-4-2-69 20038494 PMC3525207

[B2] Jannink JL Lorenz AJ Iwata H Genomic selection in plant breeding: From theory to practice Brief Funct Genomics 2010 9 166 177 10.1093/bfgp/elq001 20156985

[B3] Schefers JM Weigel KA Genomic selection in dairy cattle: Integration of DNA testing into breeding programs Anim Front 2012 2 4 9

[B4] Ingvarsson PK Street NR Association genetics of complex traits in plants New Phytol 2011 189 909 922 10.1111/j.1469-8137.2010.03593.x 21182529

[B5] Rockman MV The QTN program and the alleles that matter for evolution: All that's gold does not glitter Evolution 2012 66 1 17 10.1111/j.1558-5646.2011.01486.x 22220860 PMC3386609

[B6] Meuwissen THE Hayes BJ Goddard ME Prediction of total genetic value using genome-wide dense marker maps Genetics 2001 157 1819 1829 11290733 10.1093/genetics/157.4.1819 PMC1461589

[B7] Wiggans GR Van Raden PM Cooper TA The genomic evaluation system in the United States: Past, present, future J Dairy Sci 2011 94 3202 3211 10.3168/jds.2010-3866 21605789

[B8] Slavov GT Howe GT Adams WT Pollen contamination and mating patterns in a Douglas-fir seed orchard as measured by simple sequence repeat markers Can J For Res 2005 35 1592 1603 10.1139/x05-082

[B9] El-Kassaby YA Funda T Lai BSK Female reproductive success variation in a *Pseudotsuga menziesii* seed orchard as revealed by pedigree reconstruction from a bulk seed collection J Hered 2010 101 164 168 10.1093/jhered/esp126 20080805

[B10] Lambeth C Lee BC O'Malley D Wheeler N Polymix breeding with parental analysis of progeny: An alternative to full-sib breeding and testing Theor Appl Genet 2001 103 930 943 10.1007/s001220100627

[B11] El-Kassaby YA Cappa EP Liewlaksaneeyanawin C Klapste J Lstiburek M Breeding without breeding: Is a complete pedigree necessary for efficient breeding? PLoS One 2011 6 e25737 10.1371/journal.pone.0025737 21991342 PMC3185014

[B12] Gugger PF Gonzalez-Rodriguez A Rodriguez-Correa H Sugita S Cavender-Bares J Southward Pleistocene migration of Douglas-fir into Mexico: Phylogeography, ecological niche modeling, and conservation of 'rear edge' populations New Phytol 2011 189 1185 1199 10.1111/j.1469-8137.2010.03559.x 21118265

[B13] Earle C The gymnosperm database: *Pseudotsuga lindleyana* http://www.conifers.org/pi/Pseudotsuga_lindleyana.php

[B14] Howe GT Jayawickrama K Cherry M Johnson G Wheeler NC Breeding Douglas-fir Plant Breed Rev 2006 27 245 353

[B15] Eckert AJ Bower AD Wegrzyn JL Pande B Jermstad KD Krutovsky KV St Clair JB Neale DB Association genetics of coastal Douglas-fir (*Pseudotsuga menziesii var. menziesii*, Pinaceae). I. Cold-hardiness related traits Genetics 2009 182 1289 1302 10.1534/genetics.109.102350 19487566 PMC2728866

[B16] Rigault P Boyle B Lepage P Cooke JEK Bousquet J MacKay JJ A white spruce gene catalog for conifer genome analyses Plant Physiol 2011 157 14 28 10.1104/pp.111.179663 21730200 PMC3165865

[B17] Barker MS Dlugosch KM Reddy ACC Amyotte SN Rieseberg LH SCARF: Maximizing next-generation EST assemblies for evolutionary and population genomic analyses Bioinformatics 2009 25 535 536 10.1093/bioinformatics/btp011 19129211

[B18] Gunderson KL Whole-genome genotyping on bead arrays Meth Mol Biol 2009 529 197 213 10.1007/978-1-59745-538-1_13 19381978

[B19] Illumina Infinium genotyping data analysis: A guide for analyzing Infinium genotyping data using the Illumina GenomeStudio Genotyping Module 2010 San Diego, CA: Pub. No. 970-2007-005 (current as of 13 January 2010), Illumina Inc 1 9

[B20] Kumar S Blaxter ML Comparing *de novo* assemblers for 454 transcriptome data BMC Genomics 2010 11 571 10.1186/1471-2164-11-571 20950480 PMC3091720

[B21] Martin JA Wang Z Next-generation transcriptome assembly Nat Rev Genet 2011 12 671 682 10.1038/nrg3068 21897427

[B22] Parchman TL Geist KS Grahnen JA Benkman CW Buerkle CA Transcriptome sequencing in an ecologically important tree species: Assembly, annotation, and marker discovery BMC Genomics 2010 11 180 10.1186/1471-2164-11-180 20233449 PMC2851599

[B23] Fernandez-Pozo N Canales J Guerrero-Fernandez D Villalobos DP Diaz-Moreno SM Bautista R Flores-Monterroso A Guevara MA Perdiguero P Collada C Cervera TM Soto A Ordas R Canton FR Avila C Canovas FM Claros MG EuroPineDB: A high-coverage web database for maritime pine transcriptome BMC Genomics 2011 12 366 10.1186/1471-2164-12-366 21762488 PMC3152544

[B24] Silby MW Winstanley C Godfrey SAC Levy SB Jackson RW *Pseudomonas* genomes: Diverse and adaptable Fems Microbiol Rev 2011 35 652 680 10.1111/j.1574-6976.2011.00269.x 21361996

[B25] Frey-Klett P Garbaye J Tarkka M The mycorrhiza helper bacteria revisited New Phytologist 2007 176 22 36 10.1111/j.1469-8137.2007.02191.x 17803639

[B26] Peterson MJ Sutherland JR Tuller SE Greenhouse environment and epidemiology of grey mold of container-grown Douglas-fir seedlings Can J For Res 1988 18 974 980 10.1139/x88-149

[B27] Gordon TR Kirkpatrick SC Aegerter BJ Wood DL Storer AJ Susceptibility of Douglas fir (*Pseudotsuga menziesii*) to pitch canker, caused by *Gibberella circinata* (anamorph = *Fusarium circinatum*) Plant Pathol 2006 55 231 237 10.1111/j.1365-3059.2006.01351.x

[B28] Lorenz WW Neale DB Jermstad KD Howe GT Rogers DL Bordeaux JM Ayyampalayam S Dean JFD Conifer DBMagic: A database housing multiple *de novo* transcriptome assemblies for 12 diverse conifer species Tree Genetics & Genomes 2012 8 1477 1485 10.1007/s11295-012-0547-y23720968

[B29] Alagna F D'Agostino N Torchia L Servili M Rao R Pietrella M Giuliano G Chiusano ML Baldoni L Perrotta G Comparative 454 pyrosequencing of transcripts from two olive genotypes during fruit development BMC Genomics 2009 10 399 10.1186/1471-2164-10-399 19709400 PMC2748093

[B30] Barakat A DiLoreto DS Zhang Y Smith C Baier K Powell WA Wheeler N Sederoff R Carlson JE Comparison of the transcriptomes of American chestnut (*Castanea dentata*) and Chinese chestnut (*Castanea mollissima*) in response to the chestnut blight infection BMC Plant Biol 2009 9 51 10.1186/1471-2229-9-51 19426529 PMC2688492

[B31] Delano-Frier JP Aviles-Arnaut H Casarrubias-Castillo K Casique-Arroyo G Castrillon-Arbelaez PA Herrera-Estrella L Massange-Sanchez J Martinez-Gallardo NA Parra-Cota FI Estrada-Hernandez MG Transcriptomic analysis of grain amaranth (*Amaranthus hypochondriacus*) using 454 pyrosequencing: Comparison with *A. tuberculatus*, expression profiling in stems and in response to biotic and abiotic stress BMC Genomics 2011 12 363 10.1186/1471-2164-12-363 21752295 PMC3146458

[B32] Logacheva MD Kasianov AS Vinogradov DV Samigullin TH Gelfand MS Makeev VJ Penin AA *De novo* sequencing and characterization of floral transcriptome in two species of buckwheat (*Fagopyrum*) BMC Genomics 2011 12 30 10.1186/1471-2164-12-30 21232141 PMC3027159

[B33] Bajgain P Richardson BA Price JC Cronn RC Udall JA Transcriptome characterization and polymorphism detection between subspecies of big sagebrush (*Artemisia tridentata*) BMC Genomics 2011 12 370 10.1186/1471-2164-12-370 21767398 PMC3150299

[B34] Hsiao YY Chen YW Huang SC Pan ZJ Fu CH Chen WH Tsai WC Chen HH Gene discovery using next-generation pyrosequencing to develop ESTs for *Phalaenopsis* orchids BMC Genomics 2011 12 360 10.1186/1471-2164-12-360 21749684 PMC3146457

[B35] Schmid R Blaxter ML: annot8r: GO, EC and KEGG annotation of EST datasets BMC Bioinformatics 2008 9 180 10.1186/1471-2105-9-180 18400082 PMC2324097

[B36] Eckert AJ van Heerwaarden J Wegrzyn JL Nelson CD Ross-Ibarra J Gonzalez-Martinez SC Neale DB Patterns of population structure and environmental associations to aridity across the range of loblolly pine (*Pinus taeda* L., Pinaceae) Genetics 2010 185 969 982 10.1534/genetics.110.115543 20439779 PMC2907212

[B37] Pavy N Pelgas B Beauseigle S Blais S Gagnon F Gosselin I Lamothe M Isabel N Bousquet J Enhancing genetic mapping of complex genomes through the design of highly-multiplexed SNP arrays: Application to the large and unsequenced genomes of white spruce and black spruce BMC Genomics 2008 9 21 10.1186/1471-2164-9-21 18205909 PMC2246113

[B38] Khan MA Han YP Zhao YF Korban SS A high-throughput apple SNP genotyping platform using the GoldenGate (TM) assay Gene 2012 494 196 201 10.1016/j.gene.2011.12.001 22209719

[B39] Eckert AJ Pande B Ersoz ES Wright MH Rashbrook VK Nicolet CM Neale DB High-throughput genotyping and mapping of single nucleotide polymorphisms in loblolly pine (*Pinus taeda* L.) Tree Genet Genomes 2009 5 225 234 10.1007/s11295-008-0183-8

[B40] Bachlava E Taylor CA Tang SX Bowers JE Mandel JR Burke JM Knapp SJ SNP discovery and development of a high-density genotyping array for sunflower PLoS One 2012 7 e29814 10.1371/journal.pone.0029814 22238659 PMC3251610

[B41] Hyten DL Song Q Choi IY Yoon MS Specht JE Matukumalli LK Nelson RL Shoemaker RC Young ND Cregan PB High-throughput genotyping with the GoldenGate assay in the complex genome of soybean Theor Appl Genet 2008 116 945 952 10.1007/s00122-008-0726-2 18278477

[B42] Jones E Chu WC Ayele M Ho J Bruggeman E Yourstone K Rafalski A Smith OS McMullen MD Bezawada Warren J Babayev J Basu S Smith S Development of single nucleotide polymorphism (SNP) markers for use in commercial maize (*Zea mays L.*) germplasm Mol Breeding 2009 24 165 176 10.1007/s11032-009-9281-z

[B43] Akhunov E Nicolet C Dvorak J Single nucleotide polymorphism genotyping in polyploid wheat with the Illumina GoldenGate assay Theor Appl Genet 2009 119 507 517 10.1007/s00122-009-1059-5 19449174 PMC2715469

[B44] Rostoks N Ramsay L MacKenzie K Cardle L Bhat PR Roose ML Svensson JT Stein N Varshney RK Marshall DF Graner A Close TJ Waugh R Recent history of artificial outcrossing facilitates whole-genome association mapping in elite inbred crop varieties Proc Natl Acad Sci USA 2006 103 18656 18661 10.1073/pnas.0606133103 17085595 PMC1693718

[B45] Chao SM Dubcovsky J Dvorak J Luo MC Baenziger SP Matnyazov R Clark DR Talbert LE Anderson JA Dreisigacker S Population- and genome-specific patterns of linkage disequilibrium and SNP variation in spring and winter wheat (*Triticum aestivum* L.) BMC Genomics 2010 11 727 10.1186/1471-2164-11-727 21190581 PMC3020227

[B46] Yan JB Yang XH Shah T Sanchez-Villeda H Li JS Warburton M Zhou Y Crouch JH Xu YB High-throughput SNP genotyping with the GoldenGate assay in maize Mol Breeding 2010 25 441 451 10.1007/s11032-009-9343-2

[B47] Yuan SH Dean JFD Differential responses of the promoters from nearly identical paralogs of loblolly pine (*Pinus taeda* L.) ACC oxidase to biotic and abiotic stresses in transgenic *Arabidopsis thaliana* Planta 2010 232 873 886 10.1007/s00425-010-1224-8 20632186

[B48] Little J Higgins JPT Ioannidis JPA Moher D Gagnon F von Elm E Khoury MJ Cohen B Davey-Smith G Grimshaw J Scheet P Gwinn M Williamson RE Zou GY Hutchings K Johnson CY Wiens M Golding J van Duijn C McLaughlin J Paterson A Wells G Fortier I Freedman M Zecevic M King R Infante-Rivard C Steart A Birkett N STrengthening the REporting of Genetic Association Studies (STREGA) - An extension of the STROBE statement Plos Med 2009 6 151 163 10.1371/journal.pmed.1000022 PMC2634792 19192942

[B49] Pare G Genome-wide association studies-data generation, storage, interpretation, and bioinformatics J Cardiovasc Transl 2010 3 183 188 10.1007/s12265-010-9181-y 20560038

[B50] Hayes B Goddard M Genome-wide association and genomic selection in animal breeding Genome 2010 53 876 883 10.1139/G10-076 21076503

[B51] Grattapaglia D Resende MDV Genomic selection in forest tree breeding Tree Genet Genomes 2011 7 241 255 10.1007/s11295-010-0328-4

[B52] Sonesson AK Meuwissen THE Testing strategies for genomic selection in aquaculture breeding programs Genet Sel Evol 2009 41 37 10.1186/1297-9686-41-37 19566932 PMC2714299

[B53] Boichard D Guillaume F Baur A Croiseau P Rossignol MN Boscher MY Druet T Genestout L Colleau JJ Journaux L Ducrocq V Fritz S Genomic selection in French dairy cattle Anim Prod Sci 2012 52 115 120 10.1071/AN11119

[B54] Iwata H Hayashi T Tsumura Y Prospects for genomic selection in conifer breeding: A simulation study of *Cryptomeria japonica* Tree Genet Genomes 2011 7 747 758 10.1007/s11295-011-0371-9

[B55] Krutovsky KV Troggio M Brown GR Jermstad KD Neale DB Comparative mapping in the Pinaceae Genetics 2004 168 447 461 10.1534/genetics.104.028381 15454556 PMC1448108

[B56] Resende MDV Resende MFR Sansaloni CP Petroli CD Missiaggia AA Aguiar AM Abad JM Takahashi EK Rosado AM Faria DA Pappas GJ Kilian A Grattapaglia D Genomic selection for growth and wood quality in Eucalyptus: Capturing the missing heritability and accelerating breeding for complex traits in forest trees New Phytologist 2012 194 116 128 10.1111/j.1469-8137.2011.04038.x 22309312

[B57] Resende MFR Munoz P Acosta JJ Peter GF Davis JM Grattapaglia D Resende MDV Kirst M Accelerating the domestication of trees using genomic selection: Accuracy of prediction models across ages and environments New Phytologist 2012 193 617 624 10.1111/j.1469-8137.2011.03895.x 21973055

[B58] Howe DK Identifying candidate genes associated with cold adaptation in Douglas-fir using DNA microarrays 2006 Oregon State University, Department of Forest Science, Corvallis, OR: MS thesis

[B59] Chang S Puryear J Cairney J A simple and efficient method for isolating RNA from pine trees Plant Mol Biol Rep 1993 11 113 116 10.1007/BF02670468

[B60] Lorenz WW Alba R Yu YS Bordeaux JM Simoes M Dean JFD Microarray analysis and scale-free gene networks identify candidate regulators in drought-stressed roots of loblolly pine (*P. taeda* L.) BMC Genomics 2011 12 10.1186/1471-2164-12-264 PMC3123330 21609476

[B61] St Clair JB Mandel NL Vance-Boland KW Genecology of Douglas fir in western Oregon and Washington Ann Bot-London 2005 96 1199 1214 10.1093/aob/mci278 PMC4247077 16246849

[B62] Jaquish B Geographic variation in ten-year height growth of interior Douglas-fir in British Columbia Joint Meeting Western Forest Genetics Assoc and IUFRO Working Parties S202-05, 06, 12, and 14: Douglas-fir, contorta pine, Sitka spruce, and Abies breeding and genetic resources: 20–24 Aug 1990 1990 Olympia, WA 2.144-2.155

[B63] Zhu YY Machleder EM Chenchik A Li R Siebert PD Reverse transcriptase template switching: A SMART (TM) approach for full-length cDNA library construction Biotechniques 2001 30 892 897 11314272 10.2144/01304pf02

[B64] Zhulidov PA Bogdanova EA Shcheglov AS Shagina IA Wagner LL Khazpekov GL Kozhemyako VV Lukyanov SA Shagin DA A method for the preparation of normalized cDNA libraries enriched with full-length sequences Russ J Bioorg Chem 2005 31 170 177 10.1007/s11171-005-0023-7 15889793

[B65] Parkhomchuk D Borodina T Amstislavskiy V Banaru M Hallen L Krobitsch S Lehrach H Soldatov A Transcriptome analysis by strand-specific sequencing of complementary DNA Nucleic Acids Res 2009 37 e123 10.1093/nar/gkp596 19620212 PMC2764448

[B66] Cronn R Liston A Parks M Gernandt DS Shen R Mockler T Multiplex sequencing of plant chloroplast genomes using Solexa sequencing-by-synthesis technology Nucleic Acids Res 2008 36 e122 10.1093/nar/gkn502 18753151 PMC2577356

[B67] Ewing B Hillier L Wendl MC Green P Base-calling of automated sequencer traces using phred. I. Accuracy assessment Genome Res 1998 8 175 185 9521921 10.1101/gr.8.3.175

[B68] Parks M Cronn R Liston A Separating the wheat from the chaff: Mitigating the effects of noise in a plastome phylogenomic data set from *Pinus* L. (Pinaceae) BMC Evol Biol 2012 12 100 10.1186/1471-2148-12-100 22731878 PMC3475122

[B69] Altschul SF Madden TL Schaffer AA Zhang JH Zhang Z Miller W Lipman DJ Gapped BLAST and PSI-BLAST: A new generation of protein database search programs Nucleic Acids Res 1997 25 3389 3402 10.1093/nar/25.17.3389 9254694 PMC146917

[B70] Zhang Z Schwartz S Wagner L Miller W A greedy algorithm for aligning DNA sequences J Comput Biol 2000 7 203 214 10.1089/10665270050081478 10890397

[B71] Suzek BE Huang HZ McGarvey P Mazumder R Wu CH UniRef: Comprehensive and non-redundant UniProt reference clusters Bioinformatics 2007 23 1282 1288 10.1093/bioinformatics/btm098 17379688

[B72] Swarbreck D Wilks C Lamesch P Berardini TZ Garcia-Hernandez M Foerster H Li D Meyer T Muller R Ploetz L Radenbaugh A Singh S Swing V Tissier C Zhang P Huala E The Arabidopsis Information Resource (TAIR): Gene structure and function annotation Nucleic Acids Res 2008 36 D1009 D1014 17986450 10.1093/nar/gkm965 PMC2238962

[B73] Gene Ontology Consortium The Gene Ontology: Enhancements for 2011 Nucleic Acids Res 2012 40 D331 D335 10.1093/nar/gkr1149 19920128 PMC2808930

[B74] Kanehisa M Goto S KEGG: Kyoto Encyclopedia of Genes and Genomes Nucleic Acids Res 2000 28 27 30 10.1093/nar/28.1.27 10592173 PMC102409

[B75] Langmead B Trapnell C Pop M Salzberg SL Ultrafast and memory-efficient alignment of short DNA sequences to the human genome Genome Biol 2009 10 R25 10.1186/gb-2009-10-3-r25 19261174 PMC2690996

[B76] Li H Durbin R Fast and accurate long-read alignment with Burrows-Wheeler transform Bioinformatics 2010 26 589 595 10.1093/bioinformatics/btp698 20080505 PMC2828108

[B77] Li H Handsaker B Wysoker A Fennell T Ruan J Homer N Marth G Abecasis G Durbin R Proc GPD The Sequence Alignment/Map format and SAMtools Bioinformatics 2009 25 2078 2079 10.1093/bioinformatics/btp352 19505943 PMC2723002

[B78] Wei Z Wang W Hu PZ Lyon GJ Hakonarson H SNVer: A statistical tool for variant calling in analysis of pooled or individual next-generation sequencing data Nucleic Acids Res 2011 1 13 10.1093/nar/gkr599 PMC3201884 21813454

[B79] Fisher R Statistical Methods for Research Workers 1932 London: Oliver and Boyd

[B80] Illumina GenomeStudio Genotyping Module v1.0 User Guide: an integrated platform for data visualization and analysis 2008 Illumina Inc, San Diego, CA: Part # 11319113 (Rev. A) 168

[B81] Wigginton JE Cutler DJ Abecasis GR A note on exact tests of Hardy-Weinberg equilibrium Am J Hum Genet 2005 76 887 893 10.1086/429864 15789306 PMC1199378

